# 3-Carboxy-4-methyl-5-propyl-2-furanpropanoic acid (CMPF) induces cell death through ferroptosis and acts as a trigger of apoptosis in kidney cells

**DOI:** 10.1038/s41419-023-05601-w

**Published:** 2023-02-02

**Authors:** Jung Sun Park, Dong-Hyun Kim, Hoon-In Choi, Chang Seong Kim, Eun Hui Bae, Seong Kwon Ma, Soo Wan Kim

**Affiliations:** Department of Internal Medicine, Chonnam National University Medical School, Gwangju, 61469 Korea

**Keywords:** End-stage renal disease, Obstructive nephropathy

## Abstract

Ferroptosis is a cell death mechanism characterized by intracellular iron accumulation and lipid peroxidation. Effects of uremic toxins on ferroptosis in the kidney are not well understood. We investigated whether protein-bound uremic toxins induce ferroptosis, resulting in cell death, using the bilateral ureteral obstruction (BUO) mouse model and kidney cells. In BUO mice, we observed elevated lipid peroxidation, increased iron concentration, and decreased glutathione peroxidase 4 (GPX4) expression. Levels of transferrin receptor 1 and system Xc-, which are involved in iron transport and storage, were also elevated, while those of ferritin heavy and light chains (FHC and FLC) were reduced. Treatment of HK-2 and NRK49F kidney cells with CMPF decreased GSH levels and the expression of GPX4, FHC, and FLC, and increased levels of ROS, lipid peroxidation, and intracellular iron concentration. CMPF-induced and erastin-induced decreases in GPX4 levels and increases in Bax and cytochrome C levels were counteracted by ferrostatin-1 pretreatment. However, GPX4 mRNA levels, protein abundance, or promoter activity were not restored by Z-VAD-FMK, a multi-caspase inhibitor. These results suggest that ferroptosis induced by CMPF treatment induces apoptosis, and inhibition of ferroptosis reduces apoptosis, suggesting that ferroptosis plays a role in triggering cell death by apoptosis.

## Introduction

Bilateral ureter obstruction (BUO) blocks the tubes that carry urine from the kidneys to the bladder (ureters). When urine cannot leave the kidneys, it starts to accumulate leading to obstructive uropathy and uremia, in which toxins build up in the blood. Uremia is reversed once the toxins are filtered out by the kidneys. However, such transient stress can lead to chronic kidney disease (CKD) despite successful remission of urinary tract obstruction [1, 2]. Once progressed into CKD, kidney damage is exacerbated by several factors, including excess amount of essential minerals, other deficiencies, and exposure to toxic elements, and is often a pivotal factor in kidney disease complications. Among them, the effect of uremic toxin**s** on the kidneys and body is serious [3–5].

Uremic toxins are organic or inorganic substances that accumulate in body fluids due to kidney disease or impaired renal function and are associated with various clinical symptoms of uremic syndrome. There are currently more than 130 uremic solutes listed, which can be classified by physicochemical properties, molecular size, protein binding, and organ tropism for their adverse effects [6, 7]. Among them, CMPF shows a high protein binding rate of more than 95% and is not efficiently removed even by hemodialysis. CMPF is one of the major endogenous metabolites of furan fatty acid, but the metabolic consequences of CMPF accumulation are still unclear [8–12]

Ferroptosis is a cell death mechanism characterized by intracellular iron accumulation and lipid peroxidation [13]. Iron accumulation in patients with kidney disease is generally known to result from impaired expression of iron metabolism-related proteins or high exposure of kidney cortex to hemoglobin [14, 15]. In addition, kidney disease patients have lipid metabolism disorders due to various factors such as obesity, hyperlipidemia, metabolic acidosis, and fatty acid oxidation disorders [16, 17]. However, effects of uremic toxins on ferroptosis in the kidney are not well understood. In this study, we investigated the effects of uremic toxins on ferroptosis with respect to iron and lipid metabolism in kidney disease, and examined whether uremic toxin-induced ferroptosis causes kidney damage by promoting other types of cell death.

## Materials and methods

### Materials

Antibodies for GPX4 (ab125066), FHC (ab65080), FLC (ab69090), TfR (ab84036), xCT (ab37185), and LDHA (ab52488) were purchased from Abcam; Bax (2772), Bcl-2 (3498), LC3B (43566), ATG5 (12994), and SQSTM1/p62 (5114) from Cell Signaling. Bioss (Woburn, MA, USA) provided anti-4-hydroxynonenal (4-HNE) (bs-6313R) antibody. KIM-1 antibody was provided by Novus biologicals (Briarwood, USA). Mouse β-actin antibody (a5316), CMPF (90833), and indoxyl sulfate (I3875) were purchased from Sigma-Aldrich, Inc. P-cresyl sulfate (HY-111431) and erastin (HY-15763) were obtained from MedChemExpress. Secondary antibodies Cy3 goat anti-rabbit antibody (ab6939), Cy3 goat anti-mouse antibody (ab97035), and Alexa Fluor 488 goat anti-rabbit antibody (ab150113) used for immunofluorescence were purchased from Abcam

### Animal experiments

All methods were carried out in accordance with relevant guidelines and regulations. All experimental protocols were approved by the Animal Care Regulations (ACR) Committee of Chonnam National University Medical School (CNUH IACUC-18010, April 19, 2019). Eight-week-old male C57BL6 mice were purchased from Samtako (Korea). Mice were anesthetized with 2% isoflurane and 100% oxygen and were divided into two groups: control (*n* = 8), bilateral ureteral obstruction (BUO, *n* = 8). BUO ligated both upper portions of the ureter using 2-0 silk through an overlap incision, and the mice were sacrificed after 22 h. Blood samples were then collected from the heart for serum Cr and BUN level determination and HPLC analysis. For analysis, mouse blood was placed in a serum separation tube (SST), incubated at room temperature for 1 h, and then centrifuged at 3,000 rpm for 10 min. The left kidney was rapidly removed and processed for western blotting or fixed in 4% paraformaldehyde solution for immunohistochemistry (IHC). Right kidney was stored at −80 °C for subsequent assays. Serum BUN and creatinine in mice were measured using the Jaffe method (Olympus 5431; Olympus Optical, Tokyo, Japan).

### High-performance liquid chromatography (HPLC)

HPLC analyses were performed using a Shimadzu LC 20 A HPLC system (Shimadzu®, Marne-la-Vallée, France) connected with a RF-10AXL fluorescence detector. The chromatographic separation was performed on a Luna C18(2) column (150 × 4.6 mm, 5 µm, Phenomenex, 00F-4252-E0) protected with a C18 guard cartridge (4.0 mm × 3.0 mm, Phenomenex, AJ0-4287) at room temperature. The mobile phase, which consisted of 50 mM ammonium formate buffer (including 0.1% formic acid) and acetonitrile (20:80, v/v), was delivered at flow rate of 1 mL/min. As in the reference, the excitation and emission wavelengths were set to 280 nm and 375 nm for IS and 265 nm and 290 nm for p-CS, respectively [18, 19].

### Cell culture

Human renal proximal tubular epithelial cells (HK-2, ATCC, Manassas, VA, USA) were cultured in Dulbecco’s modified Eagle’s Medium-F-12 (DMEM-F12) (WelGene, Daegu, Korea, LM002-04) supplemented with 10% fetal bovine serum, 100 U/mL penicillin, and 100 μg/mL streptomycin at 37 °C under a humidified 5% CO_2_ atmosphere. Normal rat kidney fibroblasts (NRK-49F, ATCC®, Manassas, VA, USA, CRL-1570) were grown in DMEM medium (WelGene, Daegu, Korea, LM001-05) supplemented with 5% fetal bovine serum, 100 U/mL penicillin, and 100 μg/mL streptomycin at 37 °C under a humidified 5% CO_2_ atmosphere.

### Histologic analysis

Kidney tissues were fixed with 4% paraformaldehyde, embedded in paraffin, and cut into 4 μm-thick sections. To assess histological morphology, immunohistochemical staining was performed using indicated antibodies and horseradish peroxidase-conjugated anti-mouse or anti-rabbit IgG secondary antibodies (Dako). The stained sections were imaged with Nikon Eclipse Ni-U microscope (Tokyo, Japan). The quantitative analysis of stained sections was performed using imageJ software (National Institutes of Health, Bethesda, MD, USA).

### Western blot analysis

The cells were harvested, washed twice with ice-cold phosphate-buffered saline (PBS), and resuspended in lysis buffer. Cell extracts were prepared by brief sonication of cell pellets in RIPA buffer containing 50 mM Tris-HCl (pH 7.2), 5 mM EDTA, 150 mM NaCl, 1% Nonidet P-40, 0.1% SDS, protease inhibitor cocktail (GenDEPOT, P3100-001), and phosphatase inhibitor cocktail (GenDEPOT, P3200-001). Kidney tissue was homogenized in RIPA buffer. Cell extracts and tissue homogenates were centrifuged at 1500 × g for 20 min at 4 °C to remove cell debris. After centrifugation, supernatants containing the protein extracts were collected, and the protein concentrations were measured using a Pierce® BCA Protein Assay Kit (Pierce Biotechnology, Inc., Rockford, IL, USA). Proteins were separated on 12% sodium dodecyl sulfate-polyacrylamide gels, and transferred onto nitrocellulose membranes. The blots were blocked at room temperature for 1 h with 5% skim milk in PBS containing 0.1% Tween-20 (PBST). The blot was then incubated overnight with the primary antibody (1:2000) at 4 °C, followed by incubation with the second antibody (1:2500), and finally with anti-rabbit horseradish peroxidase-conjugated antibodies. Specific protein bands were visualized using an enhanced chemiluminescence system. Quantitative analysis of band intensity was performed using ImageJ software (National Institutes of Health, Bethesda, MD, USA).

### Quantification of mRNA

To quantify mRNA levels, RNA was isolated from HK-2 cells using TRIzol reagent (Invitrogen), and 1 μg RNA was reverse transcribed to generate cDNA using AMV Reverse Transcription System (Promega Corp., Madison, WI, USA). Quantitative PCR (qPCR) was performed using SYBR Green PCR master mix (Thermo Fisher Scientific, Austin, USA) and StepOnePlus Real-Time PCR System (Thermo Fisher Scientific, Austin, USA). Primer sequences for qPCR were as follows: hGPX4, forward, 5’-TCAGCAAGATCTGCGTGAAC-3’, reverse, 5’-GGGGCAGGTCCTTCTCTATC-3’; hGAPDH, forward, 5’-GAGTCAACGGATTTGGTCGT-3’, reverse, 5’-TTGATTTTGGAGGGATCTCG-3’.

### Measurement of reactive oxygen species (ROS) generation

Levels of intracellular ROS were assessed using 5,6-chloromethyl-2′,7′-dichlorodihydrofluorescein diacetate (CM-H2DCFDA; Invitrogen, Carlsbad, CA, USA). Cells were pretreated with 1 µM Ferritin-1 and 1 µg/ml Deferoxamine for 1 h, treated with 400 µM CMPF and incubated in DMEM-F12 serum-free medium for 6 h. Cells were washed twice with Hanks’ balanced salt solution (HBSS) and incubated with HBSS (without phenol red) containing 10 μM CM-H2DCFH-DA (C2827, Invitrogen) for 30 min at 37 °C. Images were immediately acquired by confocal microscopy on a laser-scanning microscope (LSM 510; Carl Zeiss AG) and analyzed using ImageJ (version 1.53; National Institutes of Health).

### Flow cytometry analysis

After treatment with I.S, P-CS, and CMPF uremic toxins in serum-free medium for 24 h, HK-2 and NRK49F cells were harvested, washed twice with precooled 1X PBS and resuspended in FACS binding buffer. IgG control and experimental samples were fixed with 80% methanol for 5 min, permeabilized with 0.1% PBS-Triton X-100 for 15 min, and incubated with the primary antibody (1 × 10^6^ cells in 100 μl at 1 μg/ml) at room temperature for 30 min. Then, after washing the cells twice in FACS staining buffer, the secondary antibody was diluted 1:2000 and applied at room temperature for 30 min. Cells were analyzed using a FACS Caliber flow cytometer (Becton-Dickinson, San Jose, CA, USA).

### Confocal laser microscopy

Left kidneys of BUO and control mice were collected for immunofluorescence analyses, and HK-2 cells were seeded (3 × 10^5^ cells) in a 4-well chamber, incubated at 37 °C for 24 h, and treated with 400 μM CMPF for 24 h. Tissue samples prepared through deparaffination and hydration, and the cells fixed in 4% paraformaldehyde were prepared and blocked at room temperature for 2 h. Rabbit or mouse monoclonal antibodies against KIM-1, AQP-1, GPX4, Bax, LC3B, LDHA, FHC, FLC, TfR, and System Xc- were diluted 1:100 in blocking buffer and applied at 4 °C for 24 h. After washing, the secondary antibody was diluted 1:200 in blocking buffer and applied at room temperature for 2 h, and the cells were additionally incubated with Phalloidin (Alexa Fluor 488, green fluorescence) for 30 min. After washing, coverslips were mounted onto microslides using a ProLong Gold Antifate Reagent with DAPI (Life Technologies Corporation).

### Iron content assay

The BUO mouse model and intracellular iron were assessed using an iron assay kit (Cat. No: K390-100; BioVision). After homogenization, the cells were centrifuged at 16,000× *g* for 10 min. The supernatant transferred to a new microfuge tube. Then, 50 μl of the sample was transferred into a 96-well plate, and iron assay buffer was added so that the final volume was 100 μl/well to prepare for iron (II) measurement. To measure total iron, 5 μl of iron reducing agent was added to reduce iron (III) to iron (II), and iron assay buffer was added to give a final volume of 100 μl/well. Finally, after incubation at 37 °C for 30 min, 100 µl of iron probe was added, and the mixture was incubated at 37 °C for 1 h. Absorbance was measured at 593 nm using a microplate reader. Sample readings were applied to an iron standard curve to determine the iron(II) and total iron(II + III) content of the sample. Free ferrous iron (Fe2^+^) reacts with iron probe to produce a stable colored complex with absorbance at 593 nm. Ferric iron (Fe3^+^) can be reduced to form Fe2^+^ enabling the measurement of total iron (Fe2^+^ and Fe3^+^). The level of ferric iron (Fe3^+^) was calculated by subtracting ferrous iron from total iron.

### Lipid peroxidation assay

Tissue lipid peroxidation was measured using EZ-lipid peroxidation (TBARS) assay kit (TBA200, Dogen) with MDA as a standard. Kidney tissue was prepared at 100 mg/ml in 1X PBS containing 1X Butylated Hydroxyanisole (BHT). After homogenization on ice, centrifugation was performed at 13,000 rpm at 4 °C for 5 min, and only the supernatant was transferred to a new tube. 200 μl of the supernatant sample collected from the tissue was mixed with 200 μl of the indicator solution (a solution containing 10 ml of Acid Reagent and 1 vial of indicator, Thiobarbituric Acid) in a microtube. After reacting at 65 °C for 45 min, 150 μl each was dispensed into a 96-well microplate and absorbance was measured at 540 nm using a plate reader. MDA in the sample reacts with thiobarbituric acid (TBA) to generate a MDA-TBA adduct. The MDA-TBA adduct was quantified colorimetrically. HK-2 and NRK49F cells were analyzed by FACS using lipid-peroxidation (Cell-based) assay kit (ab243377, abcam).

### Total and reduced glutathione (GSH) measurement

After washing 50 mg of mouse kidney tissue several times in cold 1X PBS, the tissue was homogenized in 1 ml of cold mamlian lysis buffer (1X PBS solution with 0.5% NP-40). After homogenization, it was centrifuged at 12,000 rpm for 15 min in a 4 °C centrifuge and the supernatant was transferred to a new tube. HK-2 and NRK49F cells were lysed by pipetting up and down 1 × 10^7^ cells into 500 μl mammalian lysis buffer. Tissue and cell samples were deproteinized using trichloroacetic acid (TCA) and neutralized using sodium bicarbonate (NaHCO3). Total and reduced GSH levels were analyzed with a fluorescence monitoring microplate reader at Ex/Em = 490/520 nm using the GSH/GSSG Ratio Detection Assay Kit II according to the manufacturer’s instructions (ab205811, abcam, Cambridge, MA, USA).

### Transient transfection and luciferase assay

HK-2 cells (5 × 10^5^) were seeded and grown until 60–70% confluence was reached. Then, 2 μg of promoter constructs and pGL3-empty vectors were transfected into cells using FuGene HD, according to the manufacturer’s protocol. The pRL-null plasmid encoding Renilla luciferase was included in all transfections to monitor the transfection efficiency. After 24 h, the media was replaced with serum free media. The cells were pretreated with 5 μM Fer-1 and 10 μM Z-VAD-FMK for 1 h, and treated with 400 μM CMPF. At 48 h post-transfection, the levels of firefly and Renilla luciferase activities were measured sequentially from a single sample using the Dual-Glo Luciferase assay system (Promega). Firefly luciferase activity was normalized to Renilla activity and the relative amount of luciferase activity in the untreated cells. The GPX4 reporter constructs were produced by BIOFACT (Yuseong-gu, Daejeon, Republic of Korea).

### Statistical analysis

GraphPad Prism 8 (GraphPad software version 8.01) was used for data analysis. Mann-Whitney test and Kruskal-Wallis test were used for single or multiple comparisons, and Dunn’s post hoc test was used to determine statistical significance. P-values less than 0.05 were considered to indicate significant differences. All experiments were performed at least three times.

## Results

### Increased levels of uremic toxins in BUO mice

**Fig. 1:**
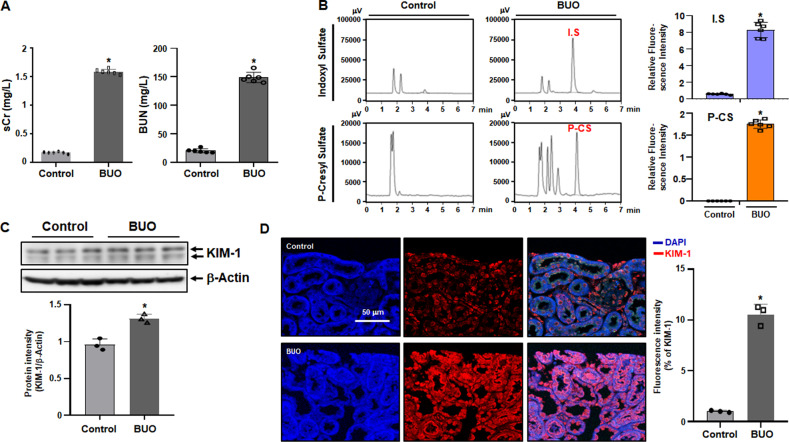
Increased levels protein-bound uremic toxins and tissue damage in bilateral ureteral obstruction (BUO) mouse kidneys. After 22 h of BUO, kidneys and serum were collected (*n* = 8). (**A**) Serum Cr and BUN levels. (**B**) The fluorescence intensity of indoxyl sulfate (IS) and p-cresyl sulfate (p-CS) as determined by chromatographic analysis. (**C**) The protein levels of Kim-1 as detected by immunoblotting. The relative protein levels are shown. The values for the control group were set to 1. (**D**) In control and BUO mouse kidneys, Kim-1 shows red immunofluorescence staining, and DAPI (4’,6-Diamidino-2-phenylindole dihydrochloride) used for nuclear staining shows blue fluorescence. The bar graph shows the fluorescence intensities with the control set to 1. Original magnification, 200x. Scale bar, 50 μm. Statistical significance was measured using Mann-Whitney test. All values are presented as the mean ± SD. *, *P* < 0.05, compared with the control.

The levels of sCr and BUN in BUO mice were significantly upregulated compared with the control group (Fig. 1A). Figure 1B shows the chromatogram for uremic toxin obtained from the serum of BUO mice. IS and p-CS levels in BUO mice were significantly higher than those in the control group. However, it was difficult to observe the CMPF peak in the chromatogram. The protein expression of KIM-1 was elevated in the kidneys of BUO mice compared with the controls (Fig. 1C). Higher KIM-1 expression in tissues of BIO mice was confirmed by immunofluorescence (Fig. 1D). These results indicate increases in tissue damage and uremic toxins levels in BUO mice.

**Fig. 2:**
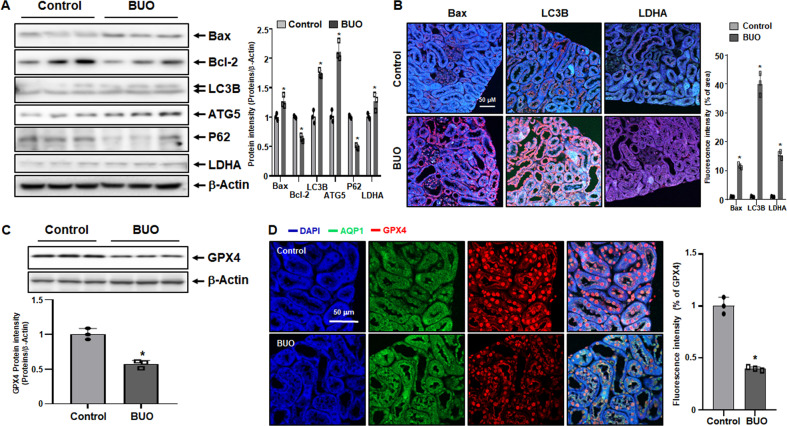
Increased cell death and decreased GPX4 expression in bilateral ureteral obstruction (BUO) mouse kidneys. (**A**) Expression levels of cell death-related proteins Bax, Bcl-2, LC3B, ATG5, P62 and LDHA in BUO and control mice (immunoblotting). The values for the control group were set to 1. (**B**) Immunofluorescence staining of Bax, LC3B, and LDHA in control and BUO mouse kidneys. The expression of cell death-related proteins are shown in red, and nuclei are marked in blue (DAPI). The bar graph on right shows the fluorescence intensity (control is set to 1). Original magnification, 200x. Scale bar, 50 μm. (**C**) Protein levels of GPX4 detected by immunoblotting. The relative protein levels are shown. The values for the control group were set to 1. (**D**) Immunofluorescence staining of GPX4 (red) in control and BUO mouse kidneys. AQP-1 (green) was used as a marker for the proximal tubule, and 4′, 6-diamidino-2-phenylindole (DAPI, blue) was used as a maker for the nucleus. The bar graph on right shows the fluorescence intensity (control is set to 1). Original magnification, 200x. Scale bar, 50 μm. All values are presented as the mean ± SD. Statistical significance was measured using Mann-Whitney test. *, *P* < 0.05, compared with the control.

We further examined the levels of proteins involved in apoptosis, autophagy, and necrosis. Protein expression levels of Bax, LC3B, ATG5, and LDHA in BUO mice were higher than those in control mice (Fig. 2A). In addition, protein levels of Bcl-2 and P62, inhibitors of apoptosis and autophagy, respectively, were lower in BUO mice. Immunofluorescence assays on kidney tissues samples also showed that Bax, LC3B, and LDHA were significantly upregulated in BUO mice (Fig. 2B).

Ferroptosis is a type of programmed cell death dependent on iron activity and characterized by the accumulation of lipid peroxides, and is genetically and biochemically distinct from other forms of regulated cell death such as apoptosis. Cell death by ferroptosis is associated with reduced expression of GPX4, a phospholipid hydroperoxidase that protects cells from membrane lipid peroxidation. Protein levels of GPX4 were significantly reduced in BUO mice (Fig. 2C). Confirming the western blot results, GPX4 immunofluorescence intensity was reduced in BUO samples, compared with controls (Fig. 2D). Overall, these findings suggest that, in BUO mice, cell death might be associated with increased lipid peroxidation.

### Increased ferroptosis in BUO mouse kidney

**Fig. 3:**
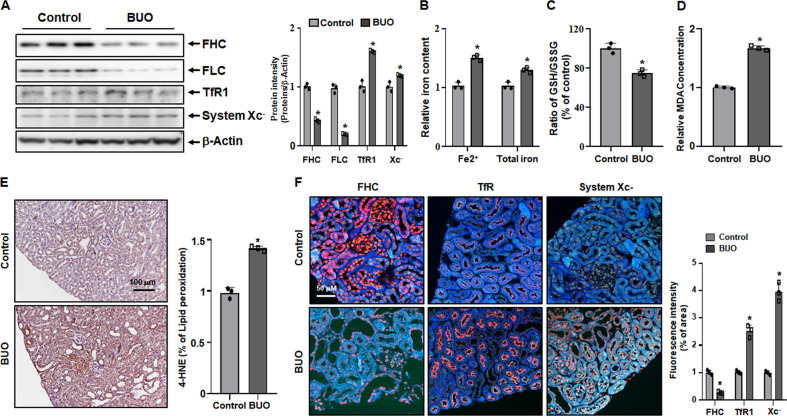
Increases ferroptosis in bilateral ureteral obstruction (BUO) mouse kidneys. (**A**) Expression of FHC (ferritin heavy chain), FLC (ferritin light chain), System Xc-, and TfR1 (transferrin receptor 1) proteins in BUO and control mice (immunoblotting). The values for the control group were set to 1. (**B**) Fe2^+^ (ferrous) and total iron (Fe2^+^ + Fe3^+^) content relative to the control. The values for the control group were set to 1. (**C**) Relative ratio of GSH/GSSG. (**D**) Relative levels of MDA concentration. (**E**) 4-HNE staining as a measure of lipid peroxidation in BUO mouse tissues. The values for the control group were set to 1. (**F**) Immunofluorescence staining of FHC, TfR1, and System Xc- (all in red) in control and BUO mouse kidneys. The nuclei are marked in blue (DAPI). The bar graph on right shows the fluorescence intensity (control is set to 1). Original magnification, 200x. Scale bar, 50 μm. All values are presented as the mean ± SD. Statistical significance was measured using Mann-Whitney test. *, *P* < 0.05, compared with the control.

Next, we investigated the induction of ferroptosis in kidney tissues of BUO mice through several experiments. Figure 3A shows the levels of proteins expressed during ferroptosis. FHC and FLC are the protein components of ferritin, which stores iron and plays a role in the maintenance of cellular homeostasis. When the cell is damaged or the limit of iron storage is exceeded due to high iron influx, the cell proceeds to ferroptosis. The protein levels of FHC and FLC significantly decreased in BUO mice. TfR1, also known as cluster of differentiation 71 (CD71), is a type II transmembrane glycoprotein that plays a role in the uptake of transferrin (Tf)-bound iron into cells. The protein expression of TfR1 was significantly elevated in BUO mice, indicating increased iron influx into the cell. System Xc-, a heterodimeric amino acid antiporter composed of a light chain xCT and a heavy chain 4F2 (4F2hc), reverse transports cystine and glutamate with a ratio of 1:1. Cysteine formed by the reduction of cystine is a rate-limiting substrate for the important antioxidant glutathione (GSH). When intracellular GSH activity is reduced, System Xc- is expressed and intracellular cystine uptake is increased. As shown in Fig. 3A, it was observed that the protein expression of System Xc- increased in BUO mice, in which ferroptosis was induced. Fe2^+^ and total iron levels were significantly upregulated in BUO mice (Fig. 3B). Next, reduced GSH and oxidized glutathione (GSSG) were measured to identify indicators of oxidative stress that can lead to ferroptosis, and potential generation of free radicals and lipid peroxide was examined. The GSH/GSH disulfide (GSH/GSSG) ratio was significantly lower in BUO mice (Fig. 3C). Lipid peroxidation was detected by measuring malondialdehyde (MDA), one of the end products of polyunsaturated fatty acid peroxidation in cells. The levels of lipid peroxidation were significantly higher in BUO samples (Fig. 3D). To further confirm the ferroptosis induction by BUO, the levels 4-hydroxynonenal (4-HNE), a marker of lipid peroxidation, and ferroptosis-related proteins were examined by IHF. The levels of 4-HNE were upregulated in BUO (Fig. 3E). FHC levels were lower, while TfR1 and System Xc- levels were higher in BUO mice compared with the control group. These results suggest that BUO induces a ferroptotic response in vivo.

### Increased lipid peroxidation upon treatment with medium-molecular-weight uremic toxin in HK-2 and NRK49F cells

**Fig. 4:**
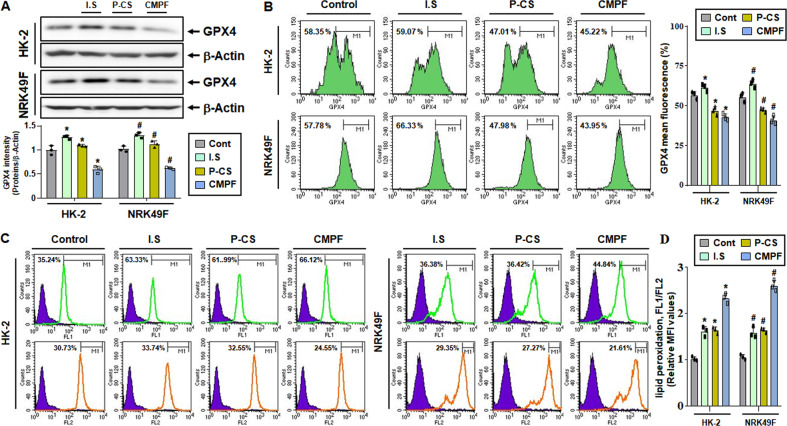
GPX4 expression and lipid peroxidation in HK-2 and NRK49F cells treated with uremic toxins. HK-2 and NRK49F cells were treated with 400 μM IS, p-CS and CMPF under serum starvation for 24 h and harvested. (**A**) GPX4 protein levels in cells treated with 400 μM uremic toxin as detected by immunoblotting. The relative protein levels are shown. (**B**) GPX4 protein levels in cells treated with uremic toxin as detected by flow cytometry. The graph on right shows the mean value of GPX4 fluorescence. (**C**) Cells treated with uremic toxins were stained with a lipid peroxidation markers for 30 min were analyzed by flow cytometry. Ex = 488 nm. Em = 530 nm (FITC) or 572 nm (PE). (**D**) The ratio of normal cells (PE) and cells with increased lipid peroxidation (FITC) is expressed as FL1/FL2. The values for the control group were set to 1. All values are presented as the mean ± SD. Statistical significance was measured using Mann-Whitney test. *, *P* < 0.05, compared with the HK-2 control. ^#^, *P* < 0.05, compared with the NRK49F control.

Next, based on the above results, we investigated in vitro whether BUO induces ferroptosis by increasing lipid peroxidation through the action of uremic toxins. Cells were treated with IS, p-CS, and CMPF, which are typical protein-bound solutes. GPX4 protein levels were lower in HK-2 and NRK49F cells treated with CMPF, compared with those treated with IS or p-CS (Fig. 4A). Decreased expression of GPX4 in HK-2 and NRK49F cells treated with uremic toxins was also confirmed by FACS analysis. It was observed that the mean fluorescence of GPX4 in cells treated with CMPF was lower than that in cells treated with IS or p-CS (Fig. 4B). The color of fluorescence signal changes from red to green when ROS-induced peroxidation takes place. We observed by FACS that green fluorescence significantly increased in the cells treated with CMPF, compared with cells treated with IS or p-CS (Fig. 4C, D). These results indicate that, among the three uremic toxins tested, CMPF induces the most profound increase in lipid peroxidation, which is thought to induce cell death by ferroptosis. Therefore, next, we examined the progression of ferroptosis upon CMPF treatment.

### Induction of ferroptosis by CMPF treatment in HK-2 and NRK49F cells

**Fig. 5:**
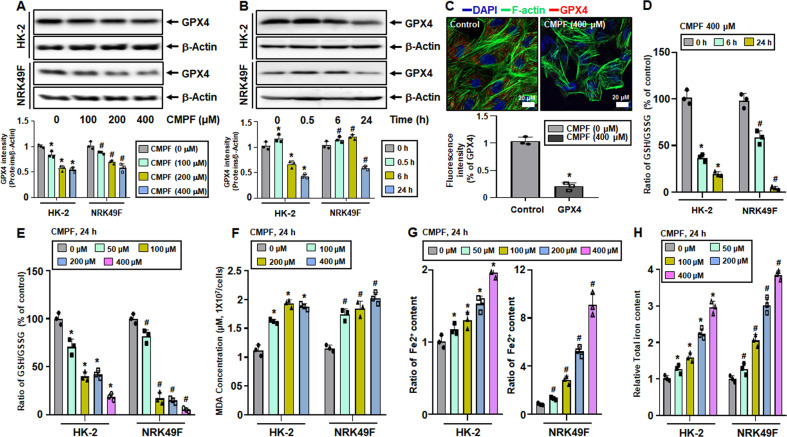
Increased ferroptosis upon uremic toxin CMPF treatment in HK-2 and NRK49F cells. HK-2 and NRK49F cells were harvested 24 h after treatment with 0–400 μM CMPF under serum starvation. (**A**) Expression of GPX4 protein in cells treated with 0, 100, 200, 400 μM of CMPF as detected by immunoblotting. The relative protein levels are shown. (**B**) Expression of GPX4 protein in cells treated with 400 μM of CMPF for 0, 0.5, 6 or 24 h, as measured by immunoblotting. The relative protein levels are shown. (**C**) Expression of GPX4 (red) detected by immunofluorescence staining in HK-2 cells after treatment (24 h) with 400 μM of CMPF. F-actin is shown in green and 4’, 6-diamidino-2-phenylindole (DAPI) in blue. The lower bar graph shows the fluorescence intensity (control is set to 1). Original magnification, 400x. Scale bar, 20 μm. (**D**) The ratio of GSH/GSSG in cells treated with 400 μM of CMPF for 0, 6, or 24 h, relative to the control. (**E**) The ratios of GSH/GSSG in cells treated with 0, 50, 100, 200, or 400 μM of CMPF for 24 h are shown compared to the control. (**F**) Relative levels of MDA concentrations compared to controls in cells treated with 0, 100, 200, or 400 μM of CMPF for 24 h. (**G**) The Fe2^+^ content in cells treated with 0, 50, 100, 200, or 400 μM of CMPF for 24 h, relative to the control. (**H**) The content of total iron (Fe2^+^ and Fe3^+^) in cells treated with 0, 50, 100, 200, or 400 μM CMPF for 24 h, relative to the control. All values are presented as the mean ± SD. Statistical significance was measured using Mann-Whitney test. *, *P* < 0.05, compared with the HK-2 control. ^#^, *P* < 0.05, compared with the NRK49F control.

**Fig. 6:**
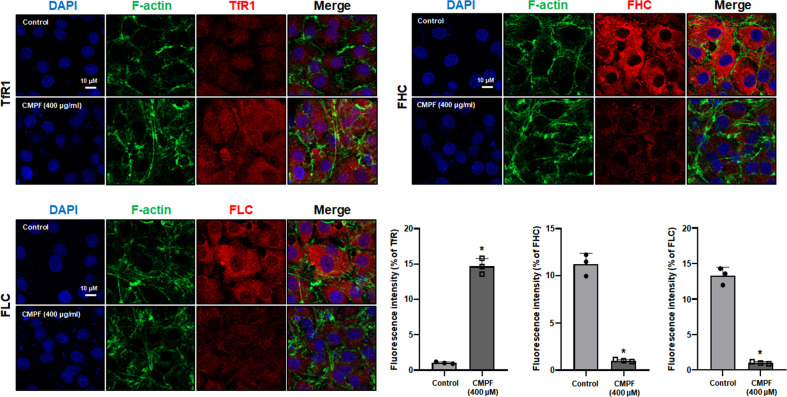
Immunofluorescence analysis of TfR1, FHC, and FLC expression after uremic toxin CMPF treatment in HK-2 cells. HK-2 cells were observed using a confocal microscope treatment with 400 μM CMPF for 24 h under serum starvation. TfR1, FHC, and FLC are indicated in red, F-actin in green, and nuclei in blue (DAPI). The bar graph shows the fluorescence intensity. All values are presented as the mean ± SD. Statistical significance was measured using Mann-Whitney test. *, *P* < 0.05, compared with the HK-2 control.

We treated HK-2 and NRK49F cells with varying doses of CMPF (0, 100, 200, or 400 μM) for 24 h and assessed GPX4 expression, lipid peroxidation, and intracellular iron levels. We also examined the time-dependent change (0, 0.5, 6, and 24 h) in these variables in cells treated with 400 μM CMPF. Figure 5A, B show that the protein expression of GPX4 was significantly reduced in HK-2 cells and NRK49F cells when 400 μM CMPF was treated for 24 h. Since CMPF is highly protein bound, the presence of albumin is of great importance. So, we co-treated CMPF with two human serum albumin concentrations, 1.75 g/dL and 4.3 g/dL, and compared GPX4 protein expression. Experiments using complete medium (with +10% FBS) were performed for comparison with serum-free conditions. As a result, when 400 μM CMPF was treated in an albumin-containing medium and a complete medium (+10% FBS), the expression of GPX4 protein was not different in the medium containing human serum albumin compared to the serum-free condition (Supplementary Fig. 1). These results suggest that ferroptosis proceeds regardless of the presence or absence of albumin. Immunofluorescence assay confirmed that that the expression of GPX4 (red) was significantly reduced in the cells treated with CMPF compared with the control (Fig. 5C). Reduced GSH and GSSG were measured after CMPF treatment in cells, and the activity of GSH/GSSG decreased the most when the cells were treated with 400 μM CMPF for 24 h (Fig. 5D, E). Intracellular lipid peroxidation increased significantly in a concentration-dependent manner after CMPF treatment for 24 h (Fig. 5F). Similarly, Fe2^+^ and total iron levels were significantly elevated upon CMPF exposure in a concentration-dependent manner (Fig. 5G, H). TfR1 levels significantly increased after CMPF treatment, while the levels of FHC and FLC were observed to be significantly decreased by immunofluorescence analysis (Fig. 6).

**Fig. 7:**
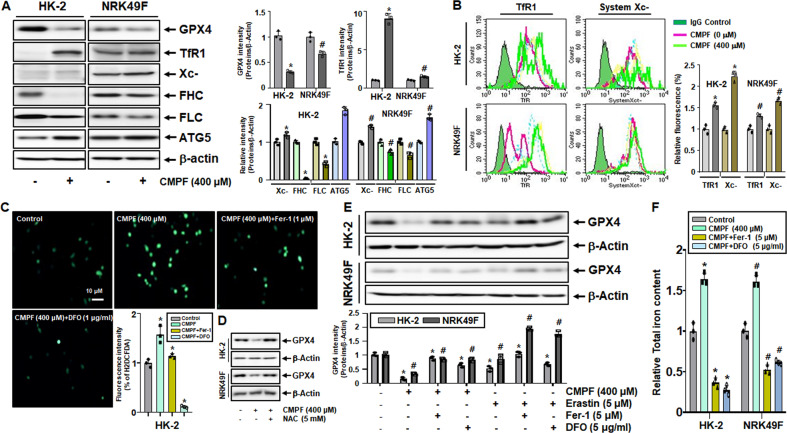
Regulation of ferroptosis-related proteins and GPX4 expression by CMPF, Erastin, Fer-1, and DFO treatment in HK-2 and NRK49F cells. HK-2 and NRK49F cells were harvested after treatment with 400 μM of CMPF or 5 μM of erastin for 24 h under serum starvation. The cells were pretreated with ferroptosis inhibitor ferrostatin-1 (Fer-1, 1 or 5 μM) and iron chelate deferoxamine (DFO, 1 or 5 μg/ml) for 1 h before the CMPF treatment. (**A**) Expression of GPX4, TfR1, System Xc-, FHC, FLC, and ATG5 proteins in cells treated with 400 μM CMPF as detected by immunoblotting. The relative protein levels are shown. (**B**) Expression of TfR1, System Xc- protein in cells treated with 0, 100, 200, or 400 μM CMPF as detected by flow cytometry. The graph on right shows the relative fluorescence values of TfR1 and System Xc- (control is set to 1). (**C**) HK2 cells were pretreated with 1 μM Fer-1 or 1 μg/ml DFO for 1 h, then treated with 400 μM CMPF, and incubated with CM-H2DCFH-DA to measure ROS levels. The bar graph shows the fluorescence intensity (control is set to 1). (**D**) After pretreatment of cells with 5 mM N-Acetylcysteine (NAC) for 1 h, the expression of GPX4 protein in cells treated with 400 μM CMPF was detected by immunoblotting. The bar graph represents the fluorescence intensity with the control set to 1. (**E**) GPX4 protein levels in cells pretreated with 5 μM of Fer-1 or 5 μg/ml of DFO for 1 h, and then treated with 400 μM of CMPF or 5 μM of erastin, as measured by immunoblotting. The relative protein levels are shown. (**F**) Relative levels of total iron content in cells treated with 400 μM CMPF, following the pretreatment with 5 μM of Fer-1 or 5 μg/ml of DFO for 1 h. All values are presented as the mean ± SD. Statistical significance was measured using Mann-Whitney test. *, *P* < 0.05, compared with the HK-2 control. ^#^, *P* < 0.05, compared with the NRK49F control.

Next, we examined whether ferroptosis was induced by CMPF treatment. Protein levels of GPX4, FHC, and FLC significantly decreased in HK-2 cells and NRK49F cells after CMPF treatment, but levels of System Xc-, TfR1, and ATG5 significantly increased (Fig. 7A). The increased expression of System Xc- and TfR1 upon CMPF treatment was confirmed by FACS analysis (Fig. 7B). CMPF-induced ROS production was significantly reduced by pretreatment with 1 μM of ferrostatin-1 (Fer-1), a ferroptosis inhibitor, and 1 μg/ml deferoxamine (DFO), an iron chelate (Fig. 7C). Cells were treated with 5 mM of the reactive oxygen species scavenger NAC (N-Acetylcysteine) to observe the expression of GPX4 protein. The protein expression of GPX4, which was reduced by CMPF treatment, was restored by NAC treatment (Fig. 7D).

The protein expression of GPX4 decreased in response to erastin treatment (5 μM), which was counteracted by pretreatment with 5 μM of Fer-1 or 5 μg/ml DFO (Fig. 7E). Total iron content increased upon CMPF treatment, and this increase was significantly attenuated in cells pretreated with Fer-1 or DFO (Fig. 7F). These findings suggest that CMPF treatment induces ferroptosis in HK-2 and NEK-49F cells.

### Regulation of apoptosis induction in CMPF-induced ferroptosis

**Fig. 8:**
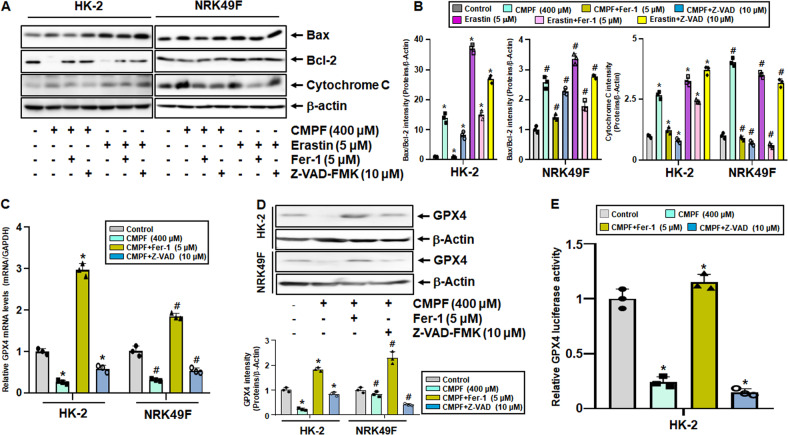
Regulation of GPX4 and apoptosis-related protein expression by CMPF, Erastin, Fer-1, and Z-VAD-FMK treatment in HK-2 and NRK49F cells. HK-2 and NRK49F cells were harvested treatment with 400 μM of CMPF or 5 μM erastin for 24 h under serum starvation. The cells were pretreated with ferroptosis inhibitor (Fer-1, 5 μΜ) or multi-caspase inhibitor Z-VAD-FMK (10 μM)1 h before CMPF or elastin treatment. (**A**) Expression levels of Bax, Bcl-2, and cytochrome C proteins as detected by immunoblotting. (**B**) Relative protein levels. (**C**) The mRNA levels of genes indicated, as measured by qRT-PCR. The values for control group are set to 1. (**D**) Expression levels of GPX4 protein in cells treated with 400 μM CMPF after pretreatment with 5 μM Fer-1 and 10 μM Z-VAD-FMK for 1 h, as measured by immunoblotting. The lower bar graph shows the relative protein levels. (**E**) GPX4 promoter activity in cells treated with 400 μM CMPF after pretreatment with 5 μM Fer-1 and 10 μM Z-VAD-FMK for 1 h, as determined by luciferase activity using a luminometer. Luciferase activity levels in the firefly/renilla control samples were set as 1. All values are presented as the mean ± SD. Statistical significance was measured using Kruskal-Wallis test followed by Dunn’s post hoc test. *, *P* < 0.05, compared with the HK-2 control. ^#^, *p* < 0.05, compared with the NRK49F control.

**Fig. 9:**
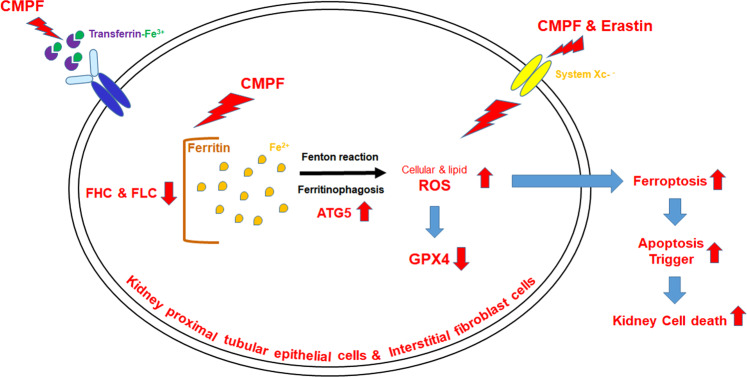
Schematic diagram of ferroptosis regulation by uremic toxin CMPF. BUO mouse model and CMPF-treated HK-2 and NRK49F cells exhibit increased levels of iron accumulation, lipid peroxidation, and ferroptosis, accompanied by decreased levels of GPX4 expression, suggesting that CMPF induces ferroptosis and acts as a trigger of apoptosis.

We further investigated whether CMPF-induced ferroptosis promoted other types cell death such as apoptosis. We examined whether the levels of apoptosis-related proteins were altered by treatment with Fer-1 or the apoptosis inhibitor Z-VAD-FMK. Treatment of HK-2 and NRK49F with 400 μM CMPF and 5 μM erastin increased Bax and cytochrome C levels and decreased BCL-2 levels; this effect was counteracted when the cells were pretreated for an hour with 5 μM Fer-1 and 10 μM Z-VAD-FMK (Fig. 8A, B). However, after Z-VAD-FMK pretreatment, erastin-treated cells show much weaker recovery compared with protein expression regulation by CMPF treatment. This result suggests that the apoptosis inhibitor is not directly involved in the progression of erastin-induced ferroptosis. The mRNA expression of GPX4, which was reduced by CMPF treatment, was increased three-fold by Fer-1 pretreatment, but was not restored by the apoptosis inhibitor Z-VAD-FMK (Fig. 8C). CMPF-induced decrease in GPX4 expression was restored by Fer-1 pretreatment, but not by Z-VAD-FMK pretreatment (Fig. 8D). Similarly, GPX4 luciferase activity decreased upon CMPF treatment, which was restored by 5 μM Fer-1 pretreatment but not by 10 μM Z-VAD-FMK pretreatment (Fig. 8E). These results suggest that ferroptosis induced by CMPF treatment also induces apoptosis, and inhibition of ferroptosis reduces apoptosis, suggesting that ferroptosis plays a role in triggering cell death by apoptosis. A schematic diagram showing that CMPF induces ferroptosis and increases cell death by acting as an apoptosis trigger based on these results is presented in Fig. 9.

## Discussion

This study demonstrated the association between uremic toxins and ferroptosis-induced cell death in the kidney, and showed that CMPF-induced ferroptosis acts as a trigger of apoptosis-like cell death, resulting in kidney damage. In BUO mouse model, increased expression of proteins related to apoptosis, autophagy, and necrosis as well as increased expression of KIM-1, a marker of kidney cell damage, were detected in response to an increase in uremic toxins. CMPF induced increases in intracellular iron and lipid peroxidation levels and a decrease in the expression of GPX4. Moreover, treatment with ferroptosis inhibitors and chelates Fer-1 and DFO reduced CMPF-induced cell death. Based on these results, this study suggests that CMPF, a protein-bound uremic toxin, induce cell death by inducing ferroptosis.

The effects of uremic toxins on the cardiovascular system and chronic kidney disease are known to be associated with cytotoxic molecules [20]. In the intestine, tryptophan and tyrosine are metabolized to indole and p-cresol by intestinal bacteria, which are then transferred to the liver through blood vessels, where they are metabolized to IS and p-CS [21, 22].

Although they have relatively low molecular weights of 212 Da and 187 Da, respectively, they have strong affinity to serum proteins, making it difficult to remove them by dialysis. It is not certain whether CMPF is produced by the intestinal metabolism of furan fatty acids generated through ingestion or it is readily present in the body. CMPF is an organic anionic uremic toxin and also has a low molecular weight of 240 Da [6, 7]. However, if dialysis fails due to strong binding to 66 kDa albumin, it may interact with active oxygen and cause cell damage. In the present study, IS and p-CS were detected by HPLC analysis, but CMPF showed no peak. It is uncertain as to why CMPF does not exhibit fluorescence intensity [23–26]. However, the evidence indicating CMPF-induced ROS and lipid peroxidation and cell damage were abundant in cell experiments. In HK-2 and NRK49F kidney cells, CMPF-induced ferroptosis was most pronounced when IS, p-CS, and CMPF were applied at the same concentrations. Various concentrations of CMPF are detected in the literature. We treated cells with high concentrations of CMPF for in vitro studies [8, 27, 28]. In our in vitro experiments, CMPF 400 μM treatment induced time-dependent and concentration-dependent decreases in GPX4 in HK-2 and NRK49F cells. In addition, even at concentrations of 100 μM and 200 μM CMPF, the protein expression of GPX4 and GSH/GSSG was decreased, while lipid peroxidation and total iron concentration increased. CMPF exposure also increased ROS levels, lipid peroxidation, and intracellular iron accumulation. However, since these toxins are highly protein bound, the presence of albumin is of great importance. So, we treated albumin and CMPF together and compared GPX4 protein expression. No significant changes were observed in GPX4 protein expression in the medium containing human serum albumin compared to serum-free conditions (Supplementary Fig. 1). Next, in order to find out how CMPF, which induces ferroptosis, increases iron accumulation inside the cell, we focused on iron transporters and receptors present on the cell membrane. CMPF treatment increased the expression levels of both TfR1 and system Xc-. TfR1, also known as CD71 (cluster of differentiation 71), is a cell membrane glycoprotein that mediates cellular uptake of iron from the plasma glycoprotein transferrin [29]. System Xc- acts as an antiporter of sodium-independent cystine and glutamate. The antiporter is a heterodimeric amino acid transporter, composed of a light chain, xCT, a heavy chain, and 4F2 as a disulfide bridge, and functions as a one-to-one reverse transport that imports the cystine and exports glutamate. Inhibition of cystine uptake into cells reduces intracellular glutathione levels, leading to ferroptosis [30, 31]. An excessive intracellular iron concentration induces ferritinophagy, a selective type of autophagy that induces ferroptosis, and decreases the expression of GPX4. It is thought that the expression of system Xc- is upregulated in order to increase the influx of cystine for GSH activity in response to the decreased expression of GPX4. Excessive intracellular iron accumulation stimulates ferritin and generates ROS based on the Fenton response, leading to lipid peroxidation and ferritinophagy. Ferritin is composed of a FHC and a FLC. Degradation of ferritin in an unstable state results in decreased expression of FHC and FLC, and increases the expression of autophagy proteins such as ATG5 and ATG7, which are expressed during the progression of ferritinophagy [32, 33]. Consistent with these, we observed increased intracellular levels of reduced Fe2^+^ in HK-2 and NRK49F cells (Fig. 5G), and increased ATG5 protein expression (Fig. 7A) by ferritinophagy progression. This result indicate that exposure to uremic toxin CMPF induces ferroptosis in kidney cells.

Ferroptosis is known as a form of cell death distinct from apoptosis, autophagy, and necrosis in terms of morphology, genetics, metabolism, and molecular biology [34, 35]. Supporting this idea, ferroptosis is not blocked by caspase-dependent apoptosis inhibitors, necrostatin-1, or RIPK1-dependent necroptosis inhibitors [36, 37]. However, our results show increased expression of proteins associated with apoptosis, autophagy, and necrosis in the BUO mouse model. In animal studies, increased ROS production by several uremic toxins, such as IS and p-CS, can lead to apoptosis, autophagy, and necrotic cell death [38–40]. In addition, in the present study, apoptosis-related protein expression was upregulated upon treatment with CMPF or erastin, and was significantly reduced when the cells were pretreated with ferroptosis inhibitor ferrostatin-1 (Fer-1) or multi-caspase inhibitor Z-VAD-FMK. Reduction in cell death by Fer-1 pretreatment suggest that System Xc- by CMPF is related to apoptotic function in kidney cells. According to a related research report, GSH levels are rapidly decreased and caspase 3-dependent apoptosis is increased in SLC7A11−/− neutrophils, and another ROS-related study reports that ER and caspase (3 and 9)-dependent apoptosis is increased in SLC7A11−/− cells [41, 42]. So, these results indicate that system Xc- itself may also act as an apoptotic pathway in kidney cells. Then, if system Xc- increased apoptosis by CMPF treatment, GPX4 expression should be restored by Z-VAD-FMK, a multiple caspases activity inhibitor. However, the present study showed that CMPF-induced reductions in levels of GPX4 mRNA, protein, and promoter activity were restored by Fer-1 treatment, but not by Z-VAD-FMK. These results show that CMPF induces ferroptosis in kidney cells, causes cell death, and also acts as a trigger of apoptosis.

In conclusion, CMPF, a protein-bound uremic toxin, induces kidney damage by acting as a ferroptosis and apoptosis trigger. This study may facilitate the development of targeted therapeutic agents for kidney disease caused by uremic toxin.

## Supplementary information

Supplementary Figure 1

Reproducibility checklist

Immunoblotting Raw Data

## Data Availability

The datasets used or analyzed during the current study are available from the corresponding author on reasonable request.
