# Supplementary material for: 3-Carboxy-4-methyl-5-propyl-2-furanpropanoic acid (CMPF) induces cell death through ferroptosis and acts as a trigger of apoptosis in kidney cells
Source: Cell Death Dis. 2023 Feb 2;14(2):78. doi: 10.1038/s41419-023-05601-w (PMC9894909; doi:10.1038/s41419-023-05601-w)
Supplement: Supplementary file 1 — Supplementary Figure 1 [file 41419_2023_5601_MOESM1_ESM.pdf]

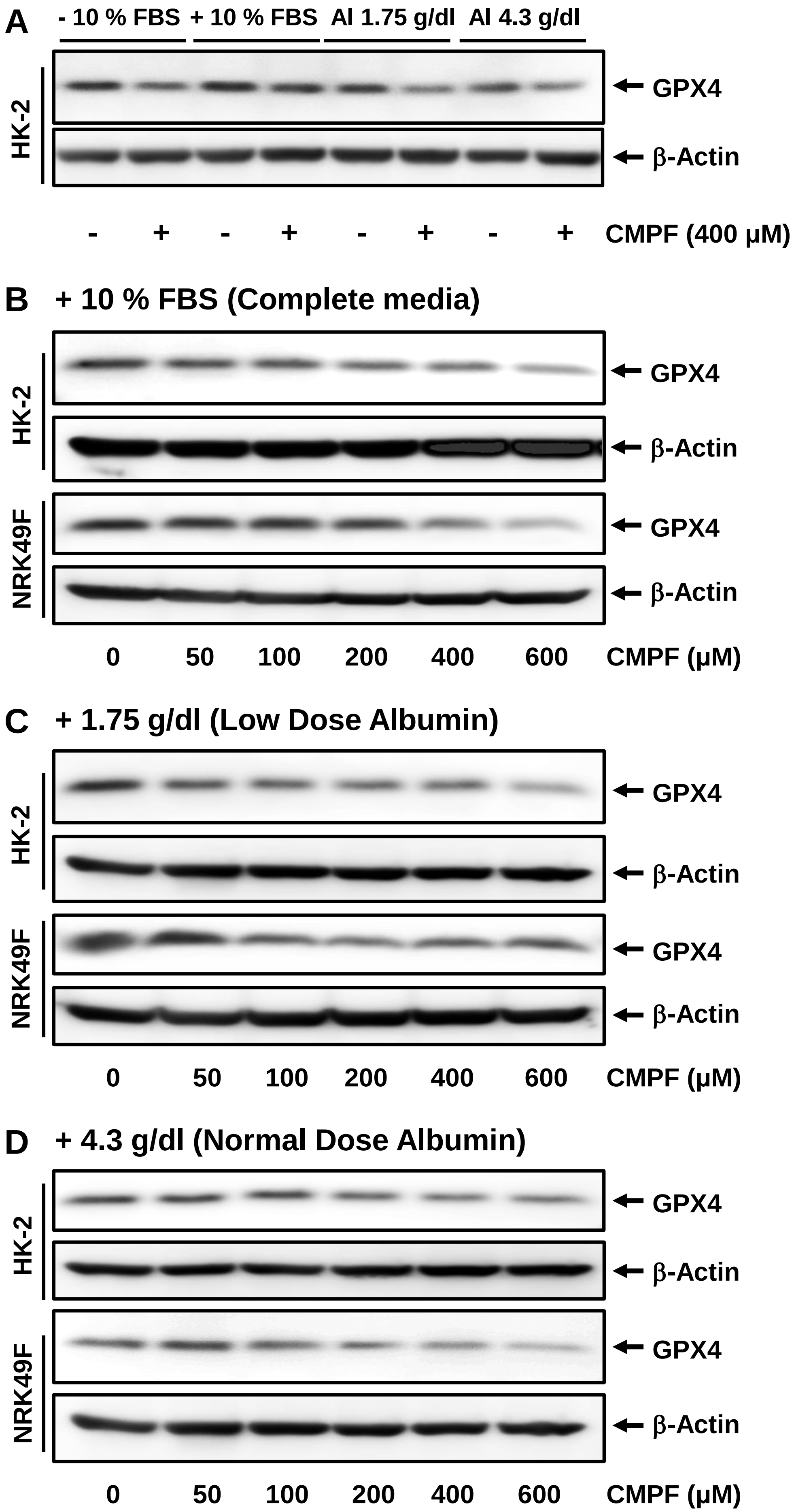

A
- 10 % FBS
+ 10 % FBS
Al 1.75 g/dl
Al 4.3 g/dl
HK-2
GPX4
β-Actin
- + - + - + - +
CMPF (400 μM)
B
+ 10 % FBS (Complete media)
HK-2
NRK49F
GPX4
β-Actin
GPX4
β-Actin
0 50 100 200 400 600
CMPF (μM)
C
+ 1.75 g/dl (Low Dose Albumin)
HK-2
NRK49F
GPX4
β-Actin
GPX4
β-Actin
0 50 100 200 400 600
CMPF (μM)
D
+ 4.3 g/dl (Normal Dose Albumin)
HK-2
NRK49F
GPX4
β-Actin
GPX4
β-Actin
0 50 100 200 400 600
CMPF (μM)

Supplementary Fig 1. GPX4 protein expression by CMPF treatment. (A) GPX4 protein expression in HK-2 cells treated with 400 μM CMPF for 24 h was reduced in all culture conditions. (B) In HK-2 and NRK49F cells, GPX4 protein expression was decreased in a dose-dependent manner in response to 0, 50, 100, 200, 400, and 600 μM concentrations of CMPF under complete media (+10% FBS) conditions. (C) In human serum albumin (1.75 g/dL, low concentration), GPX4 protein expression was decreased in a CMPF concentration-dependent manner. (D) In the medium containing human serum albumin (4.3 g/dL, normal concentration), GPX4 protein expression was reduced in a CMPF concentration-dependent manner.
