# Supplementary material for: 3-Carboxy-4-methyl-5-propyl-2-furanpropanoic acid (CMPF) induces cell death through ferroptosis and acts as a trigger of apoptosis in kidney cells
Source: Cell Death Dis. 2023 Feb 2;14(2):78. doi: 10.1038/s41419-023-05601-w (PMC9894909; doi:10.1038/s41419-023-05601-w)

Fig 1 – raw data

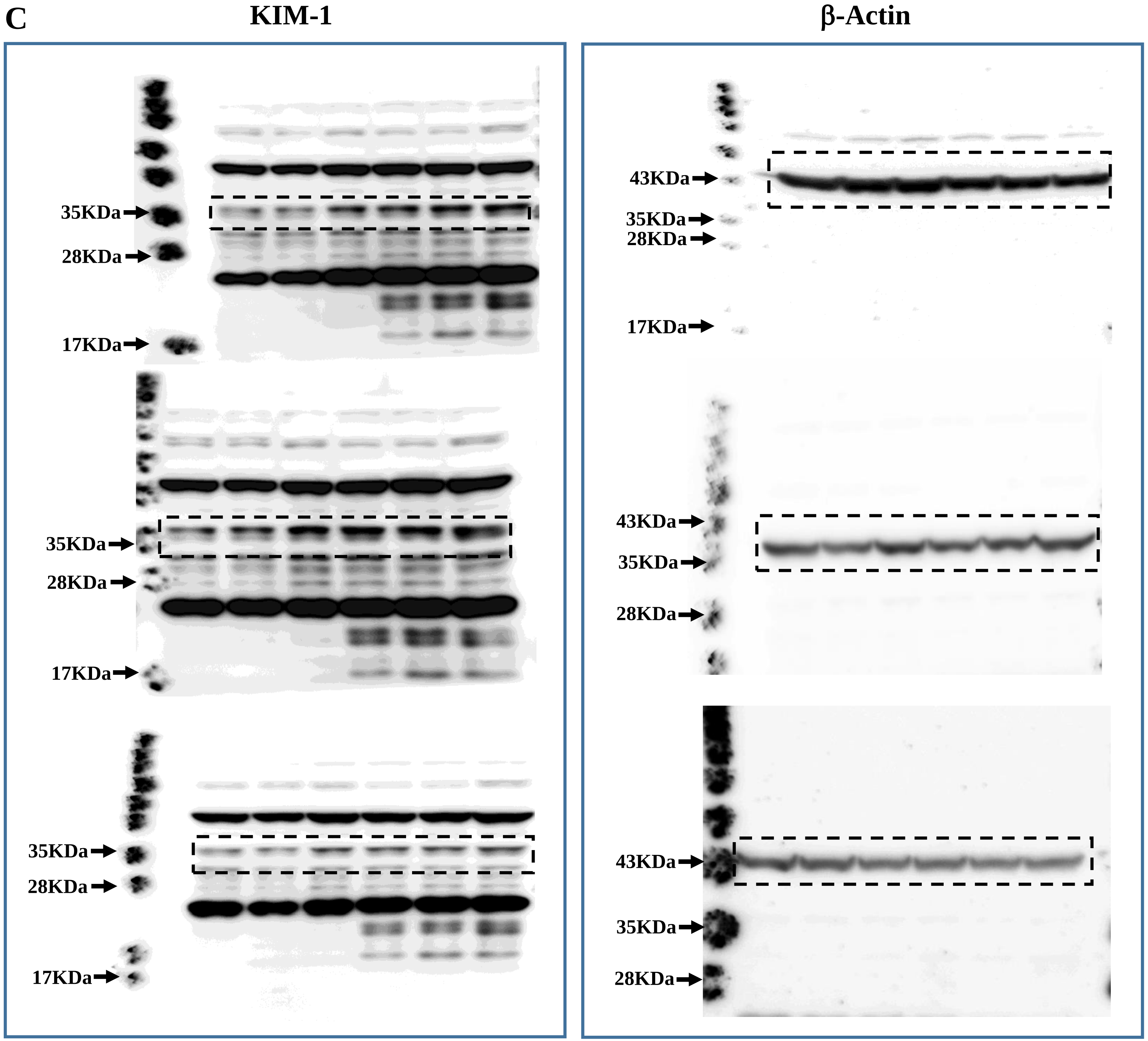

Fig 2 – raw data

A

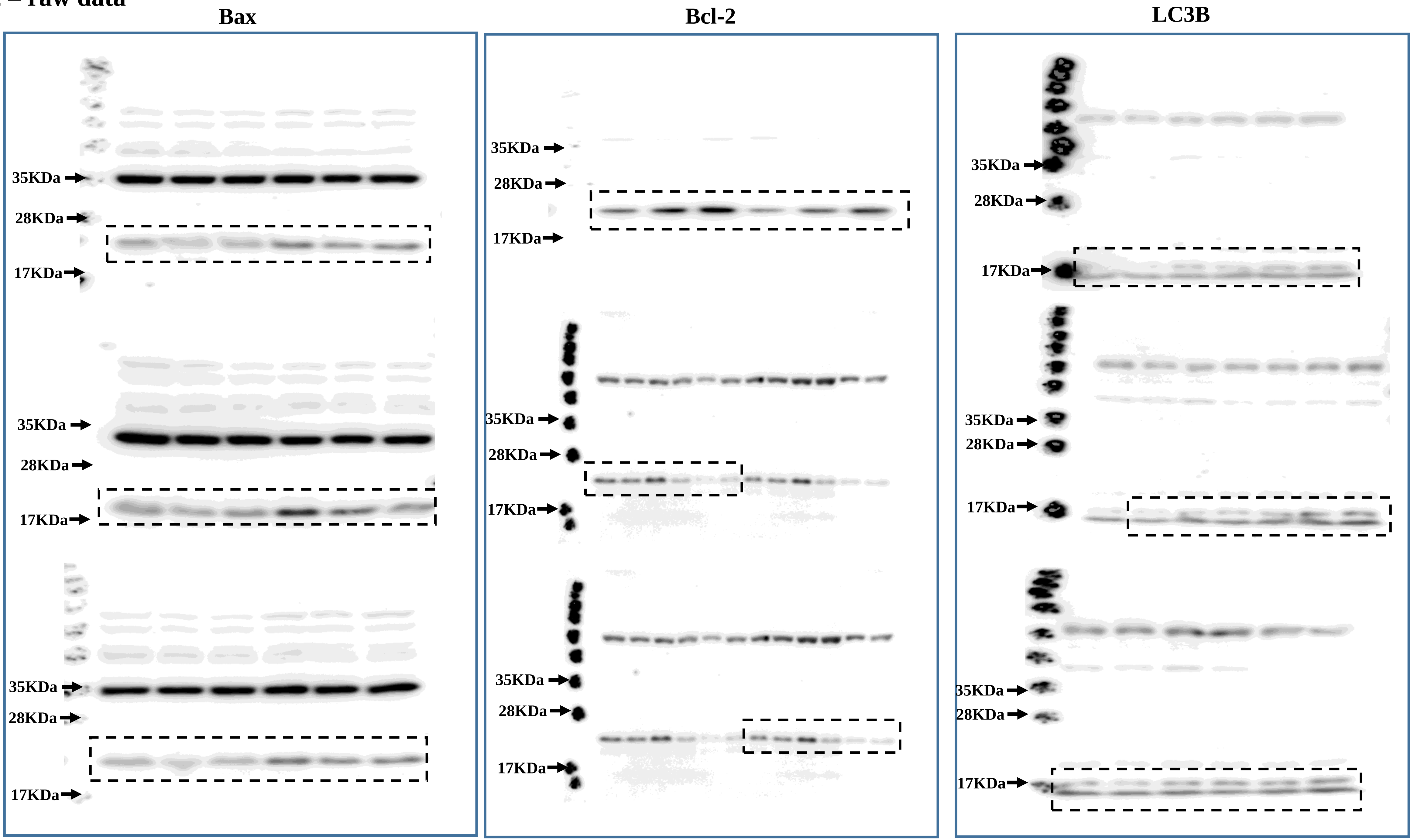

Fig 2 – raw data

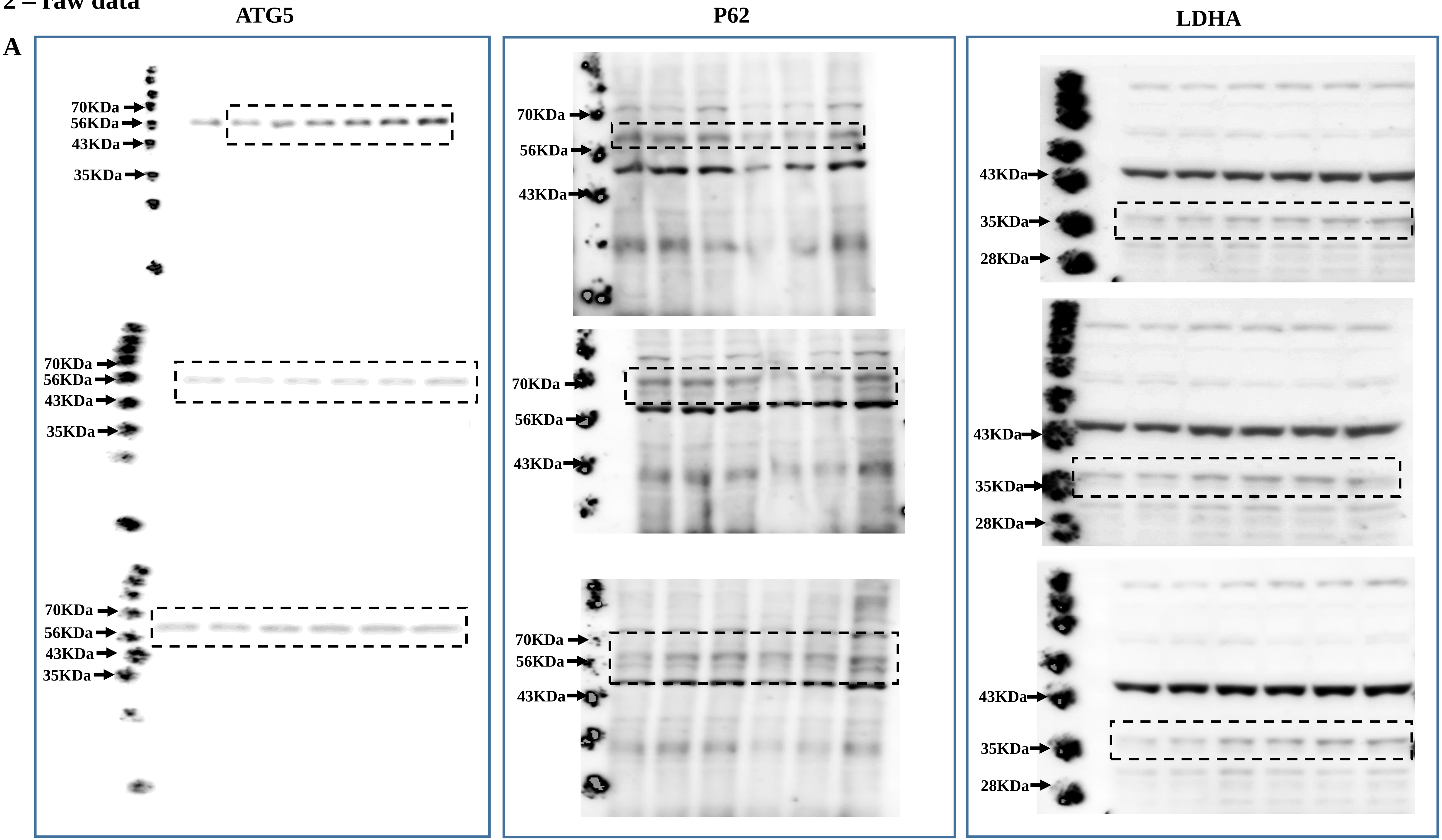

Fig 2 – raw data

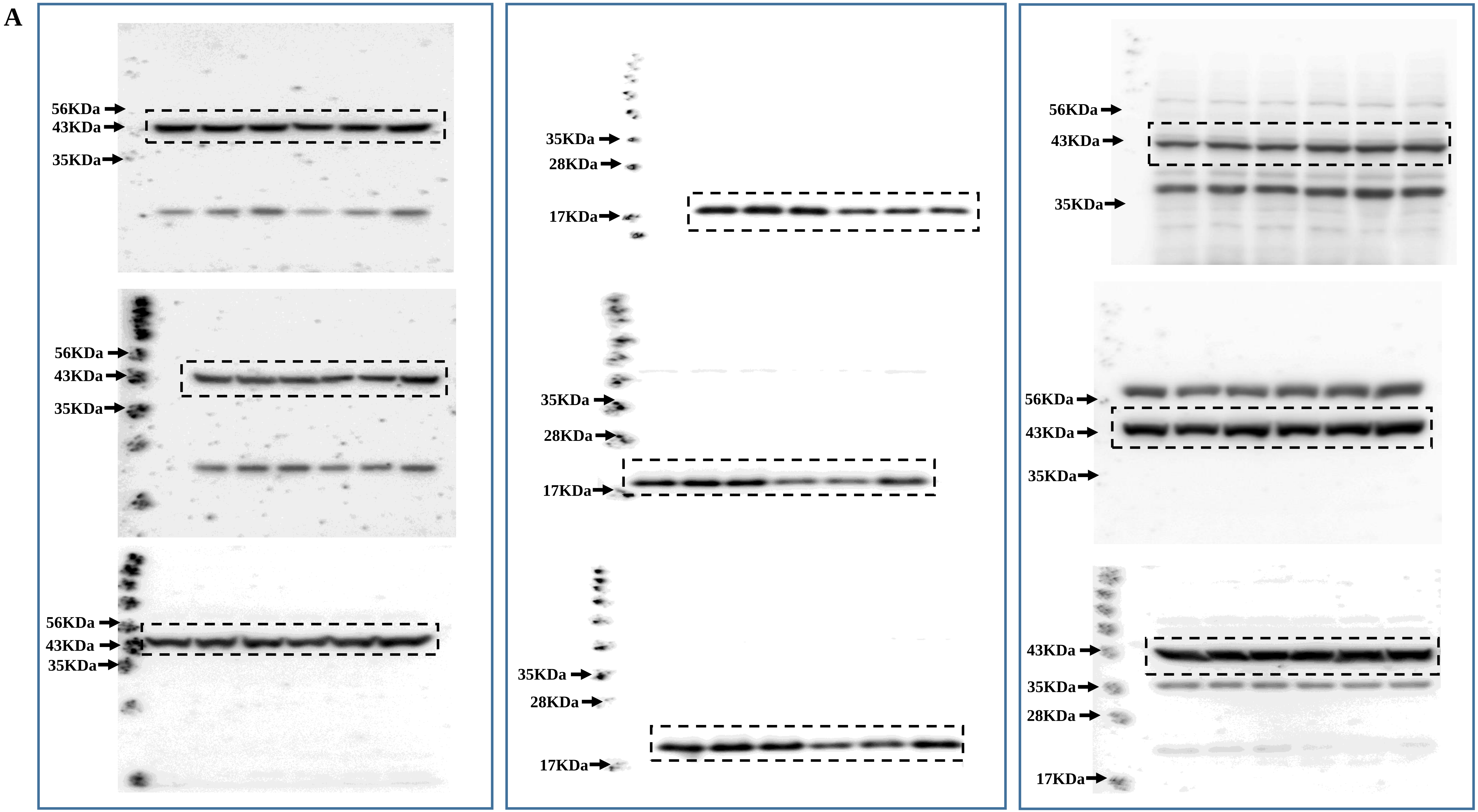

Fig 3 – raw data

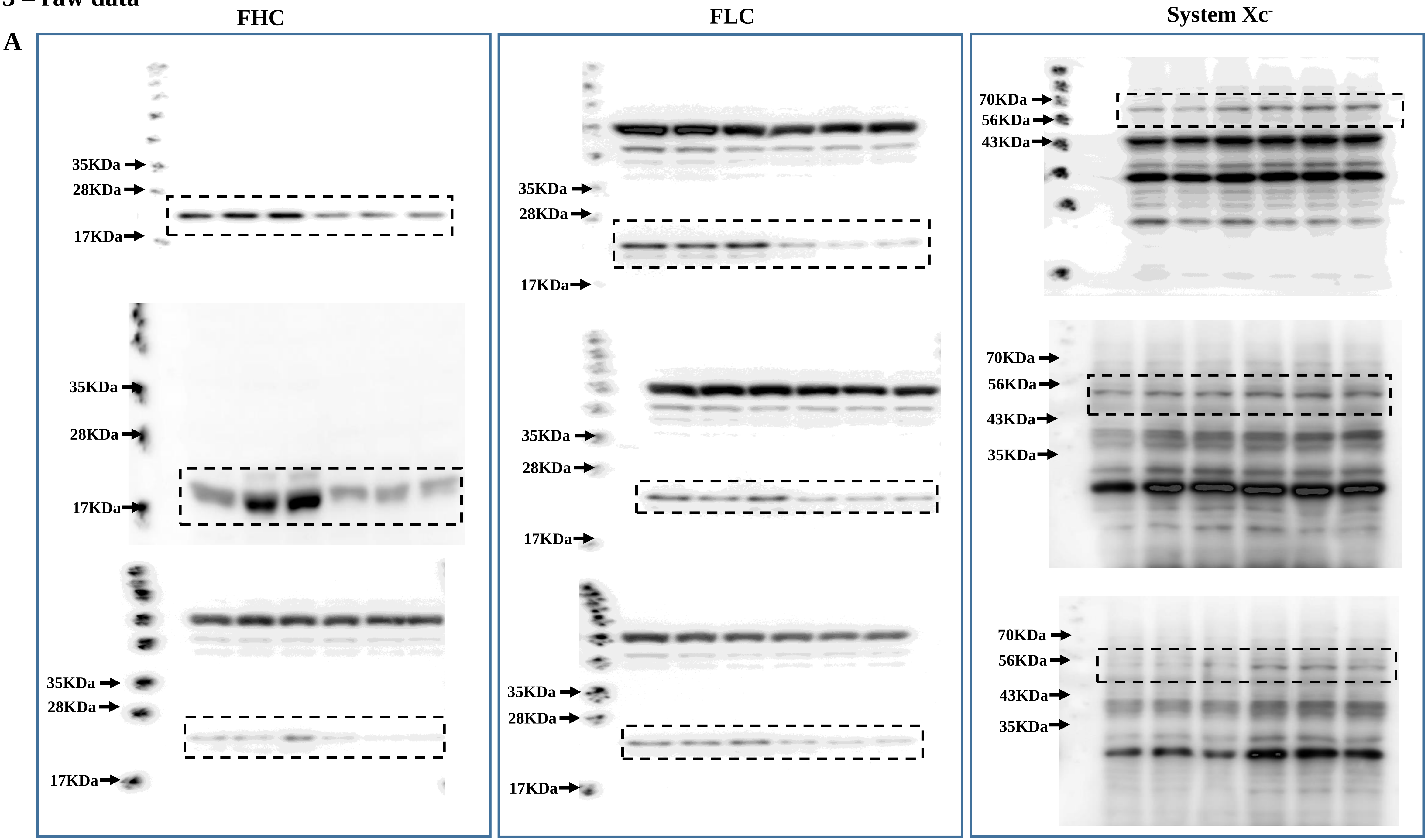

Fig 3 – raw data

A

TfR1

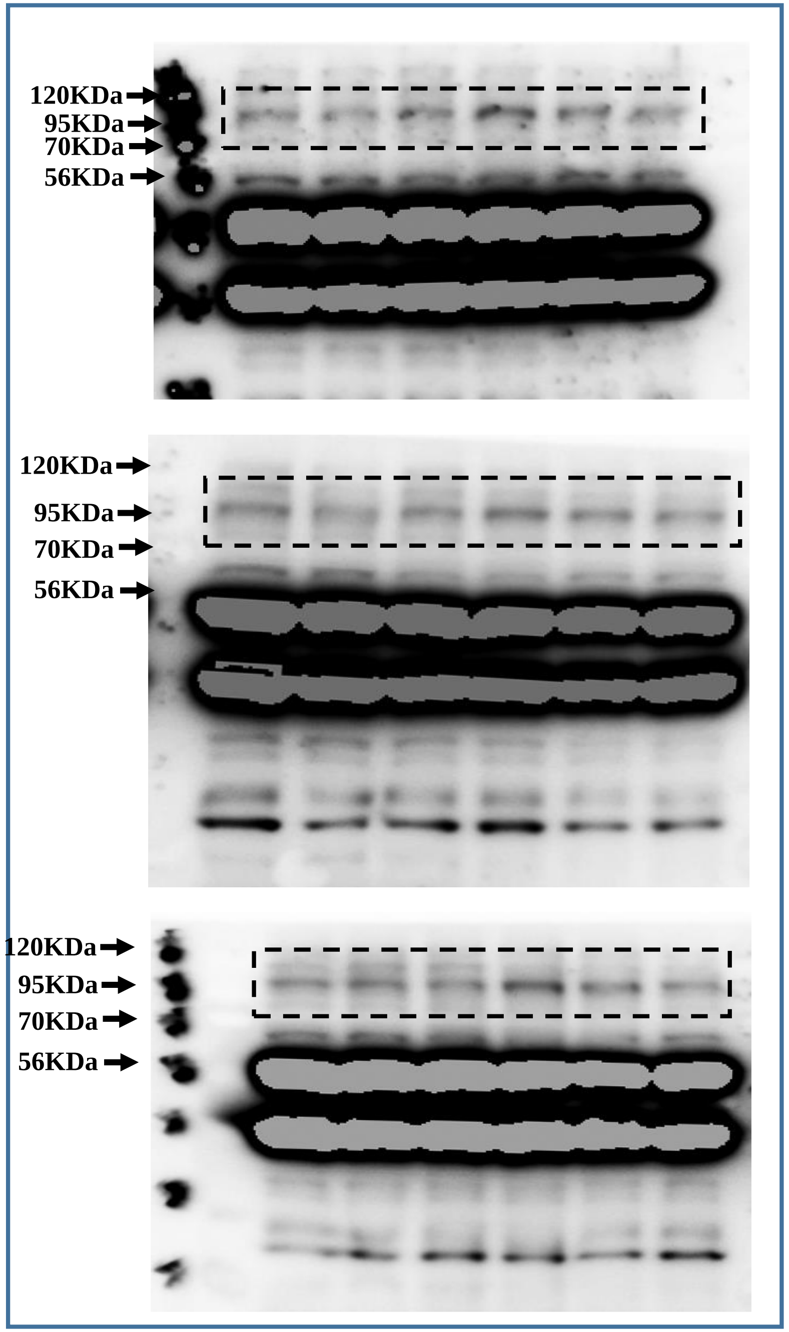

β-Actin

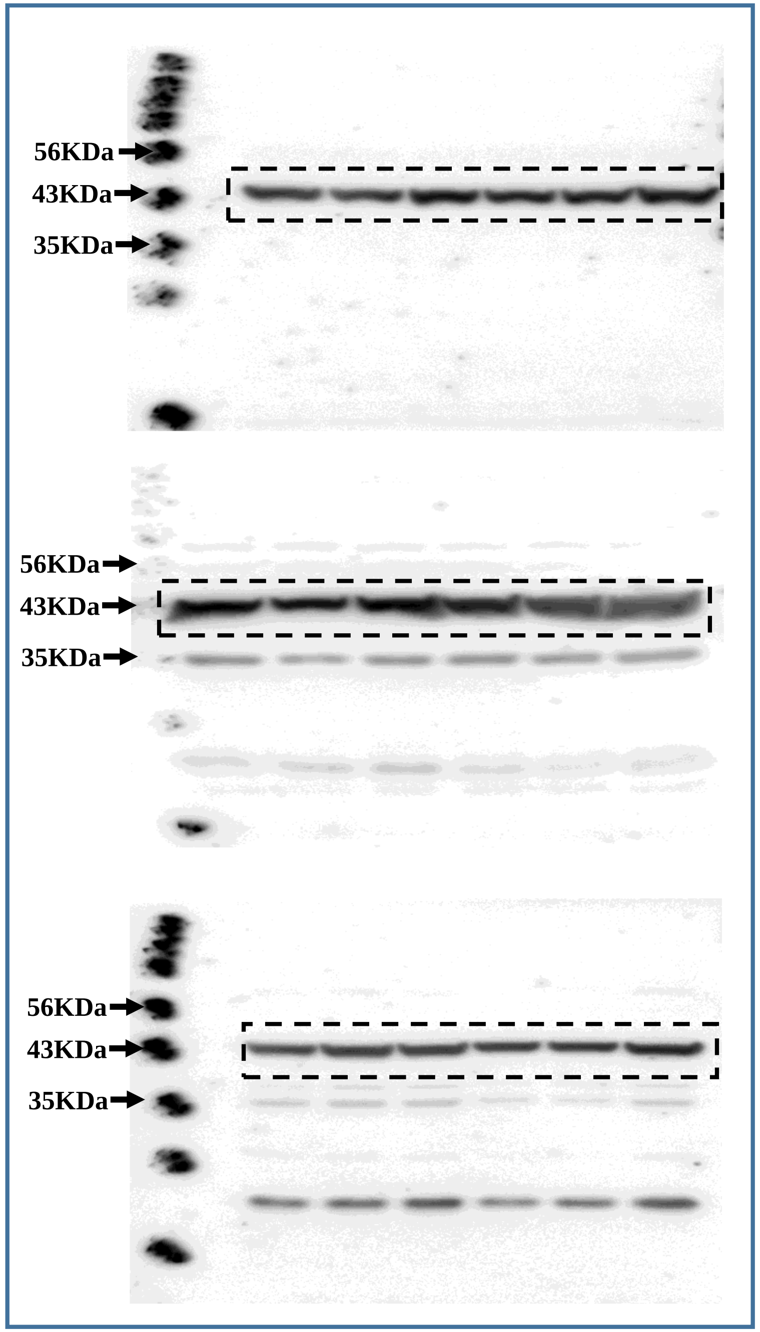

Fig 4 – raw data

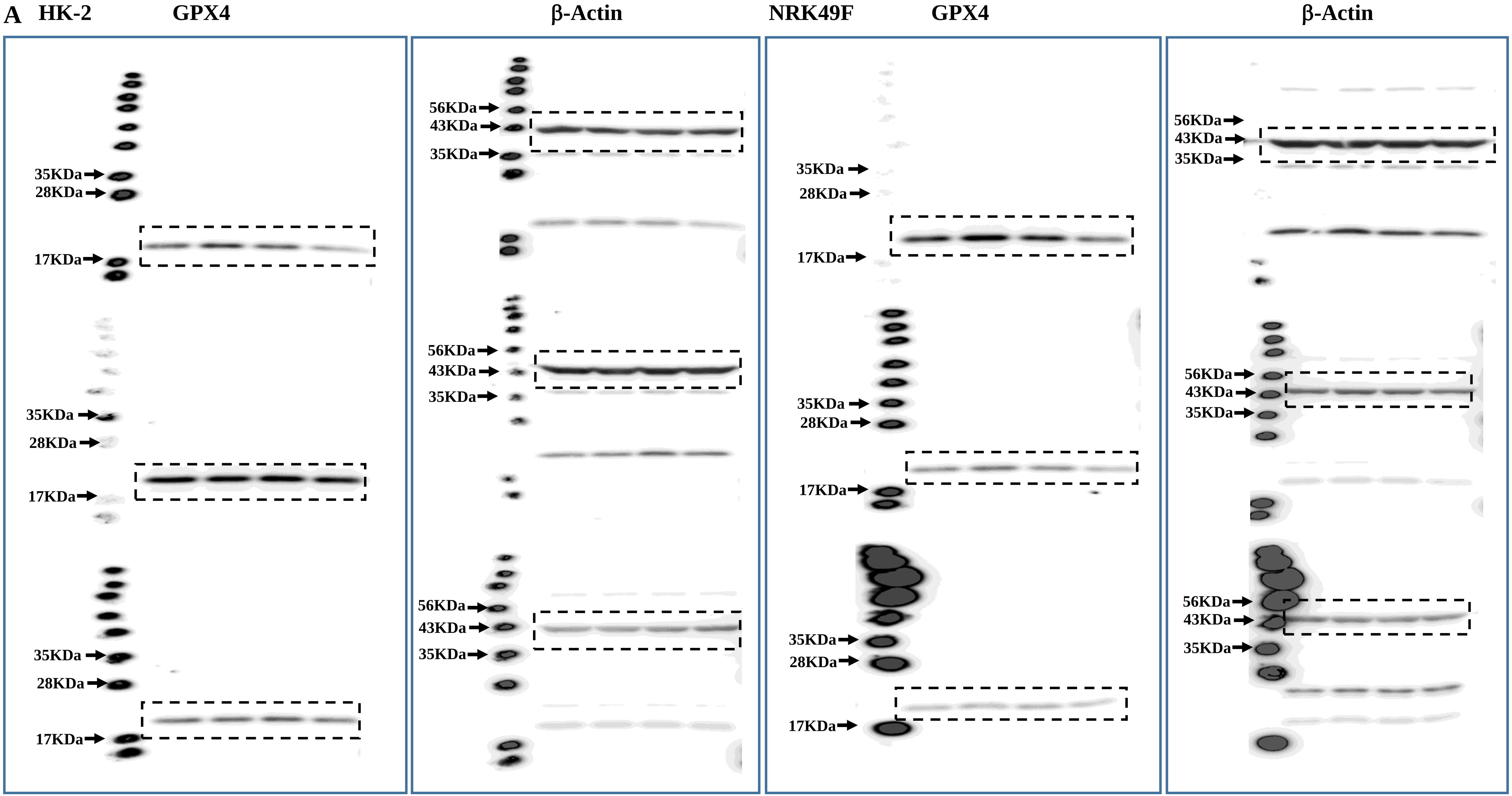

Fig 5 – raw data

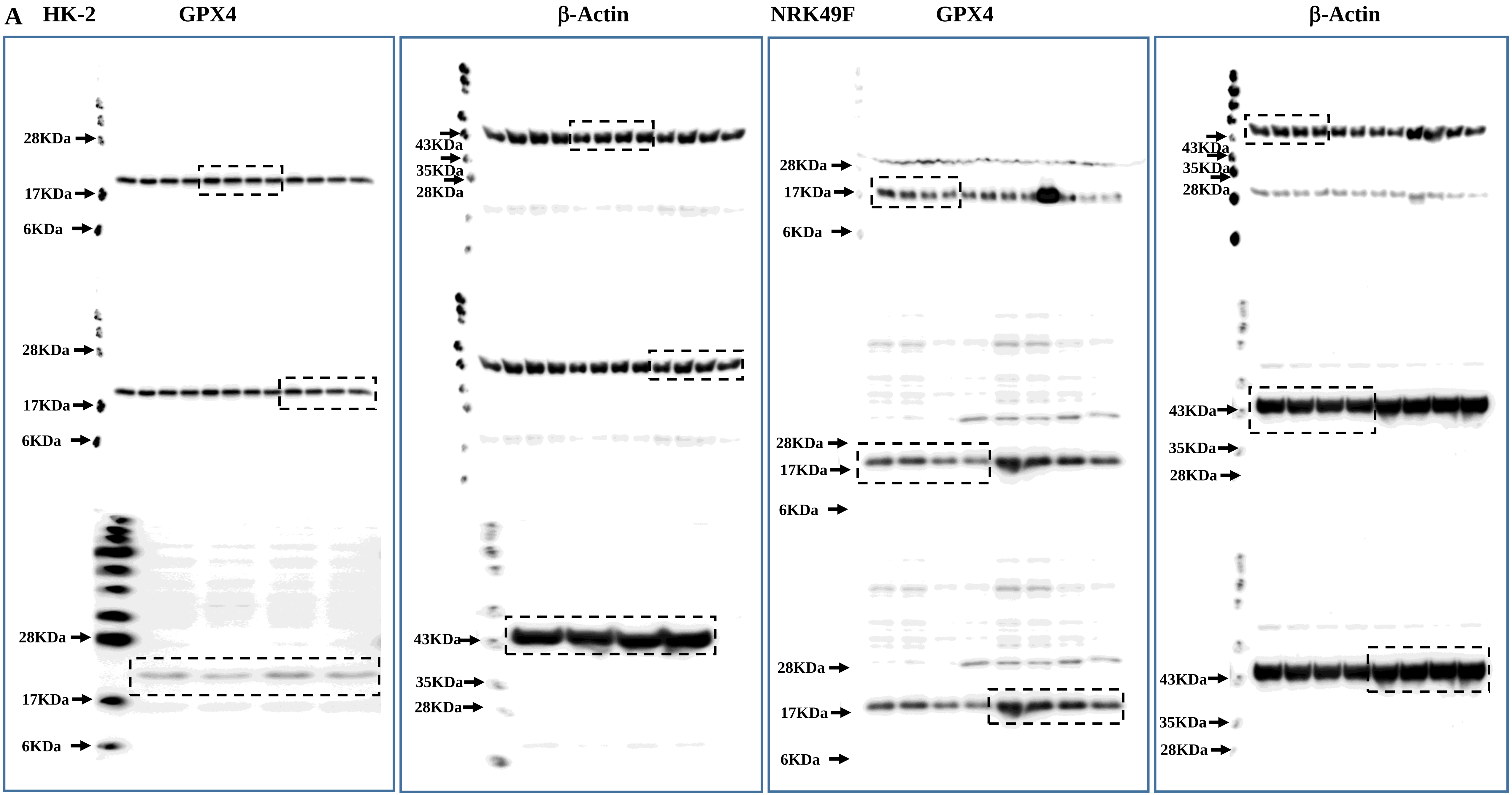

Fig 5 – raw data

B HK-2 GPX4 β-Actin NRK49F GPX4 β-Actin

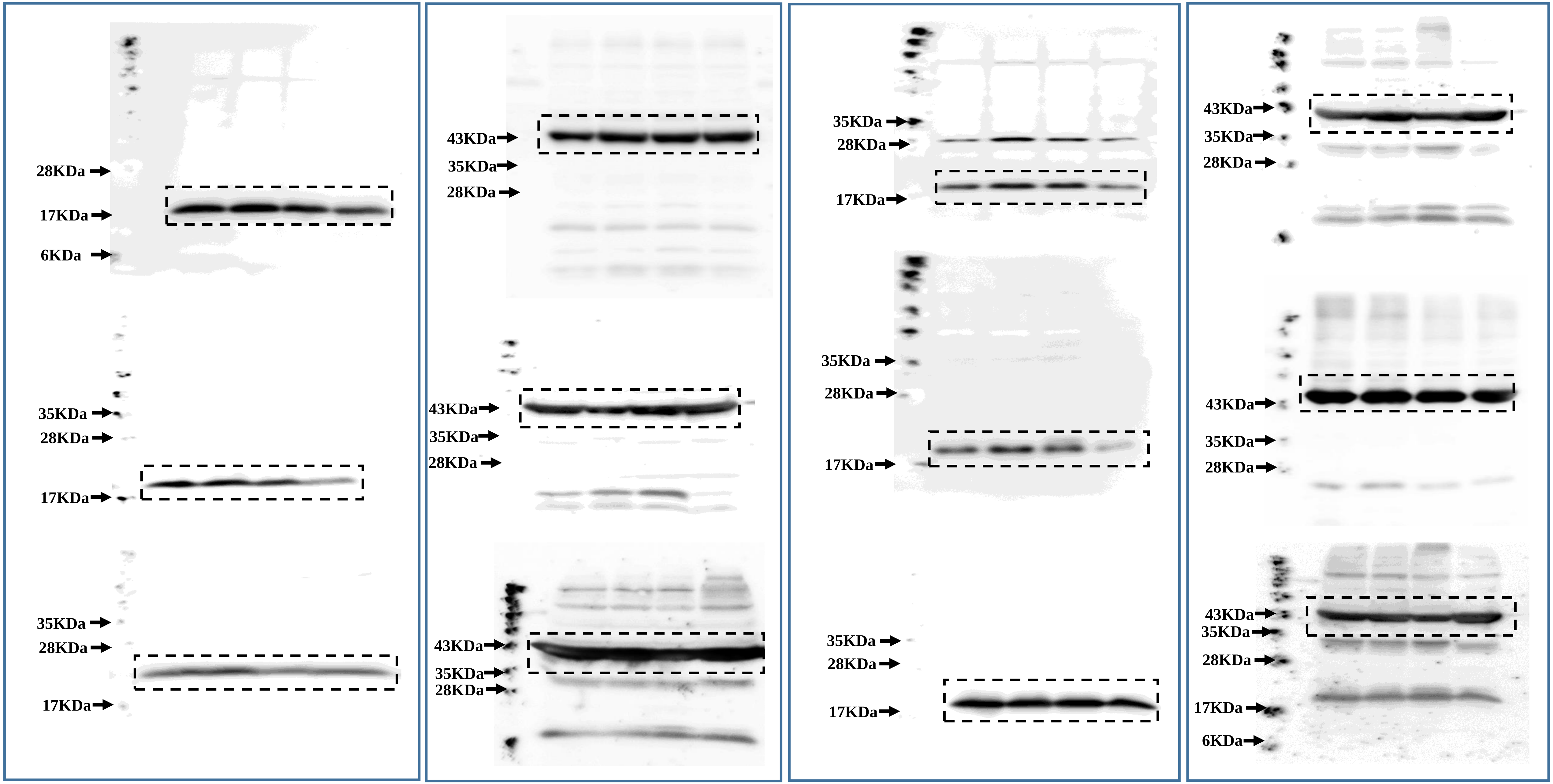

Fig 7 – raw data

A HK-2

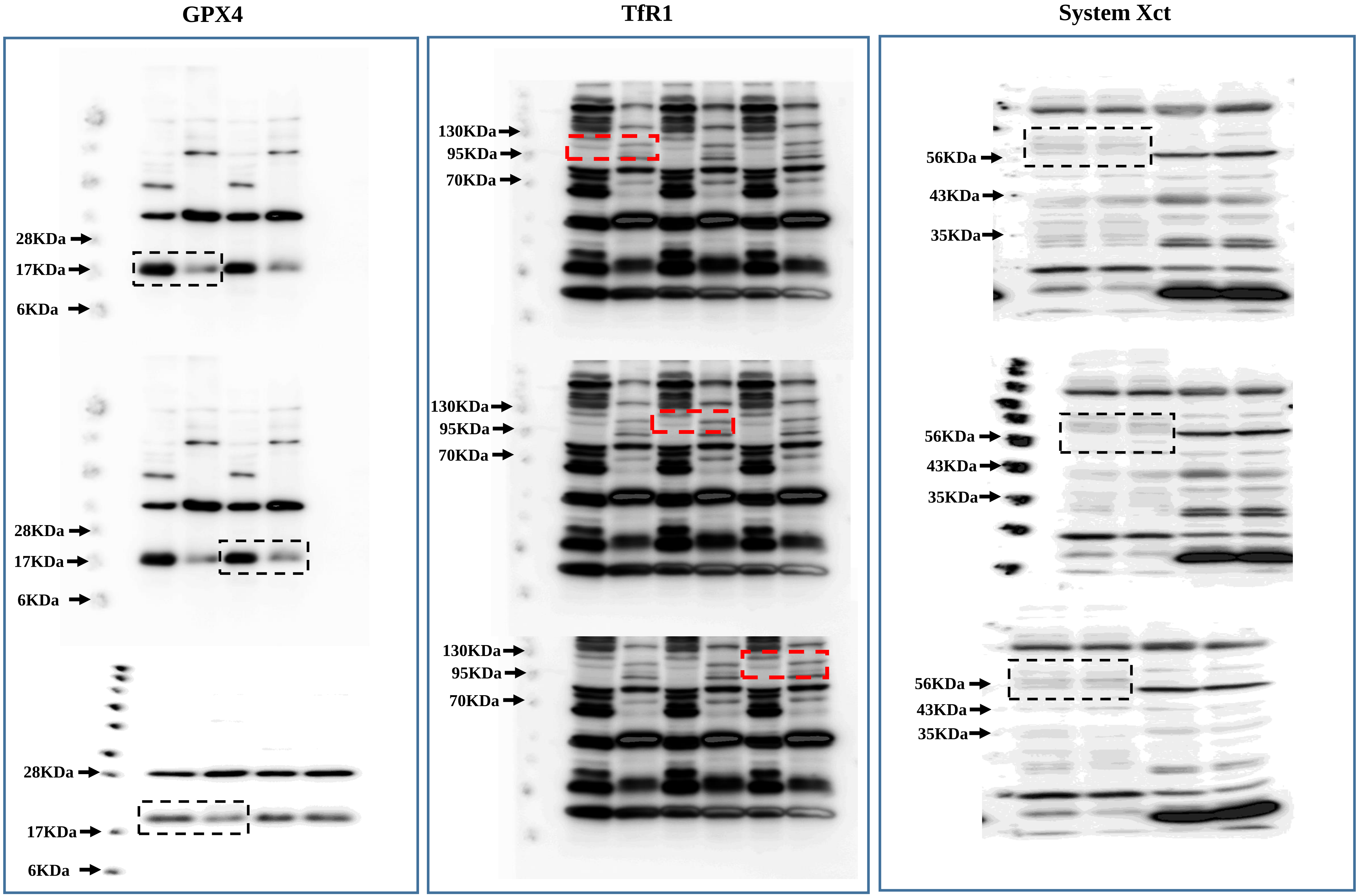

Fig 7 – raw data

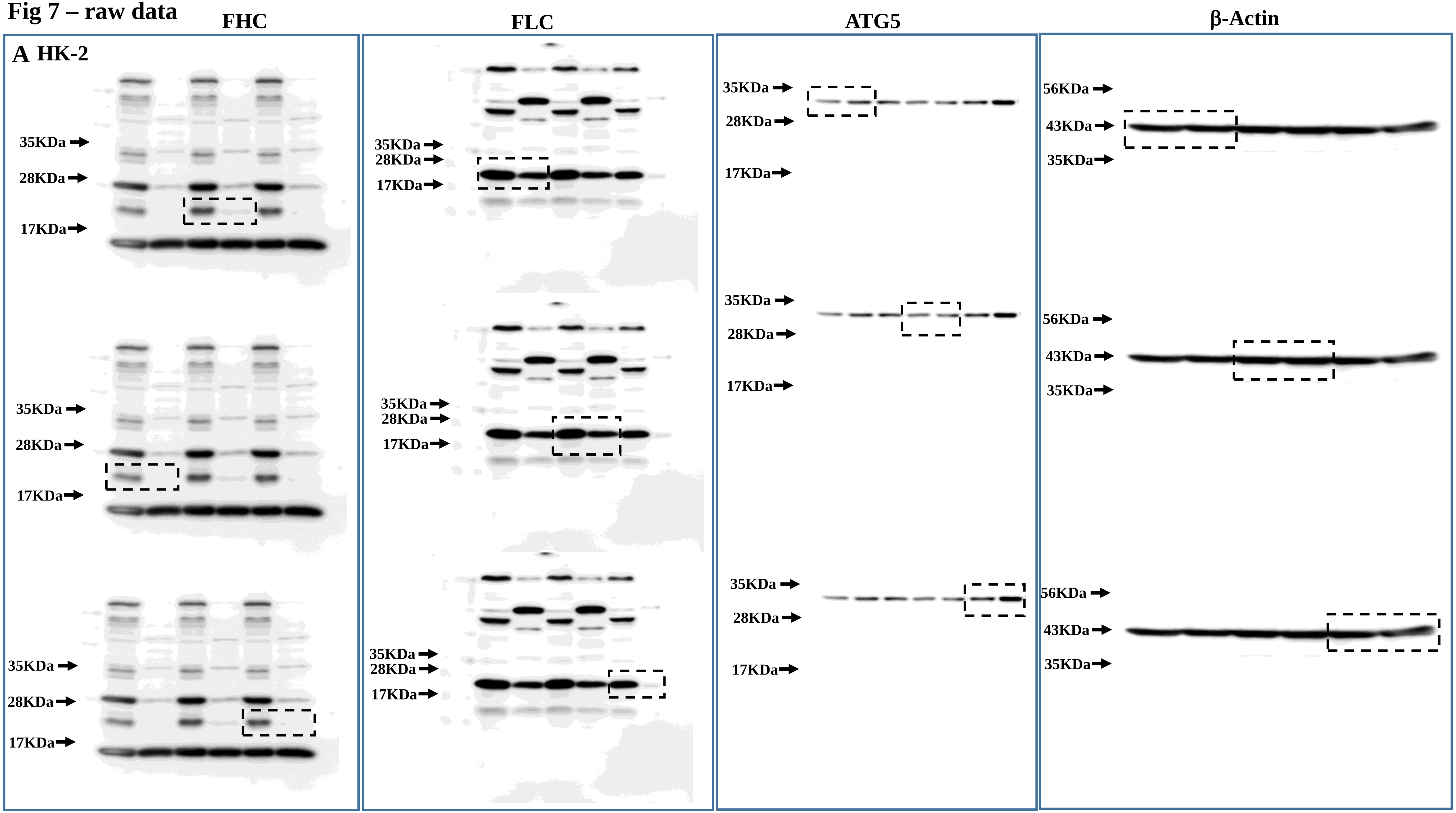

Fig 7 – raw data

A NRK49F

GPX4 TfR1 System Xct

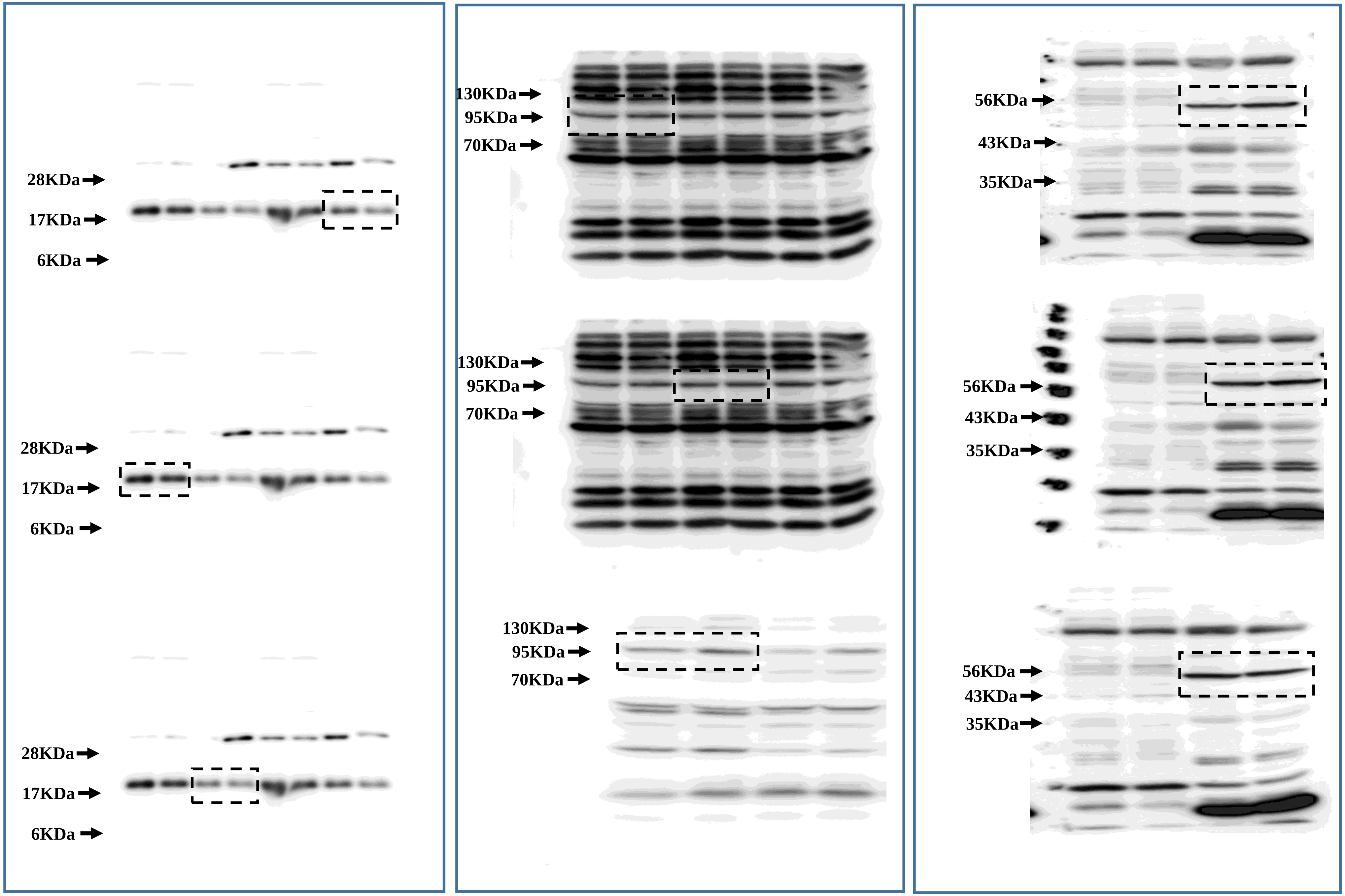

Fig 7 – raw data

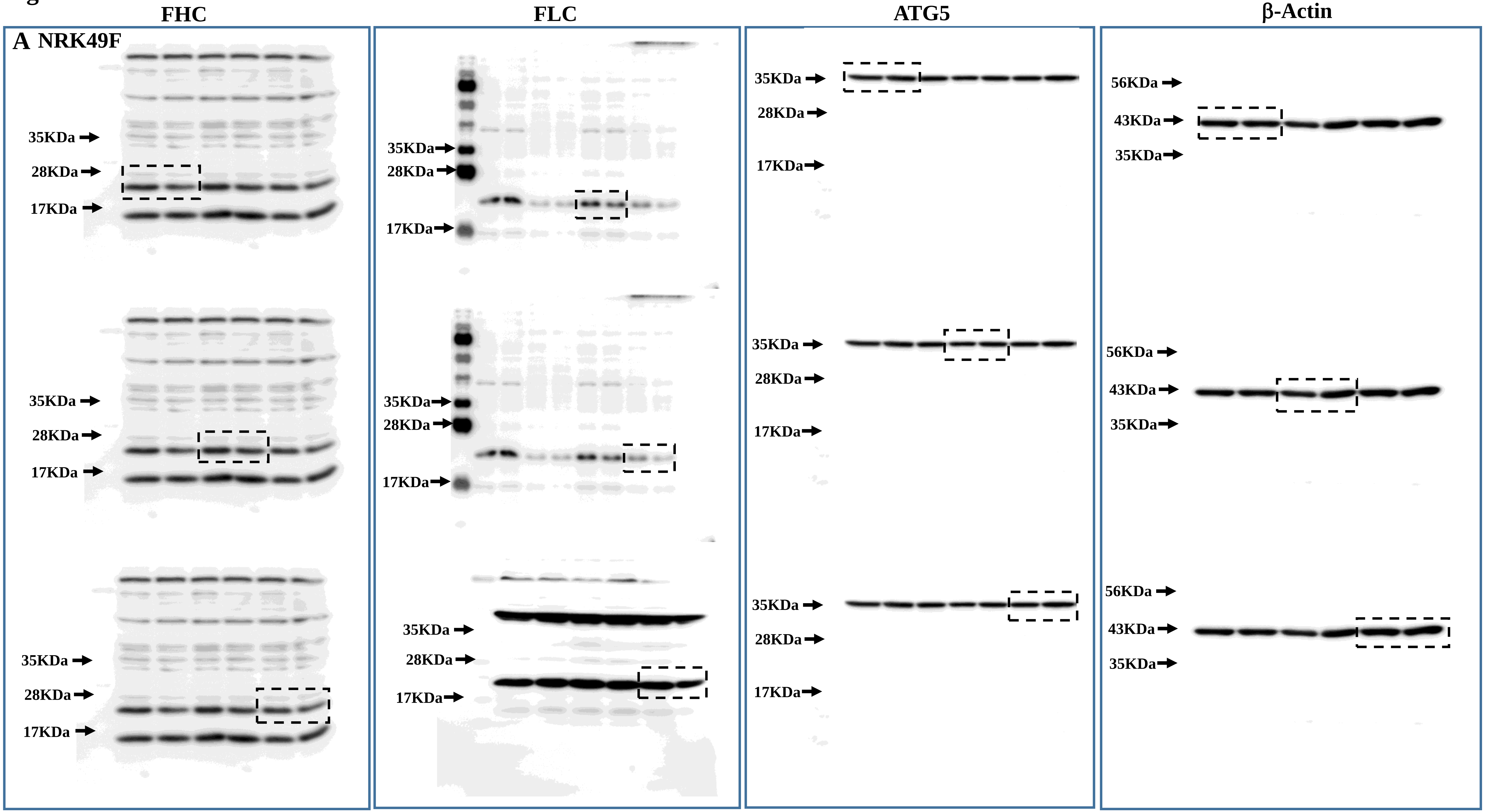

Fig 7 – raw data

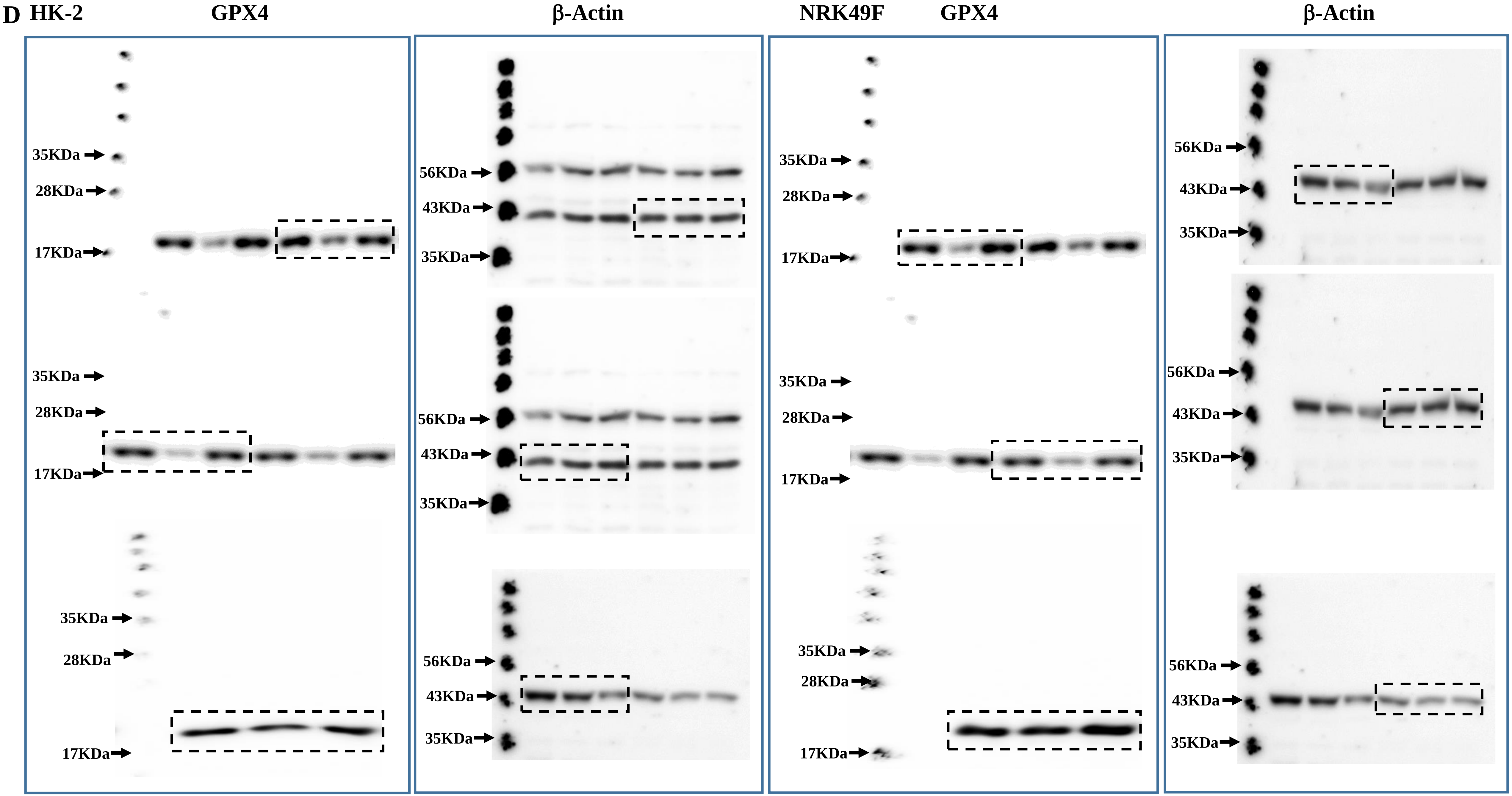

Fig 7 – raw data

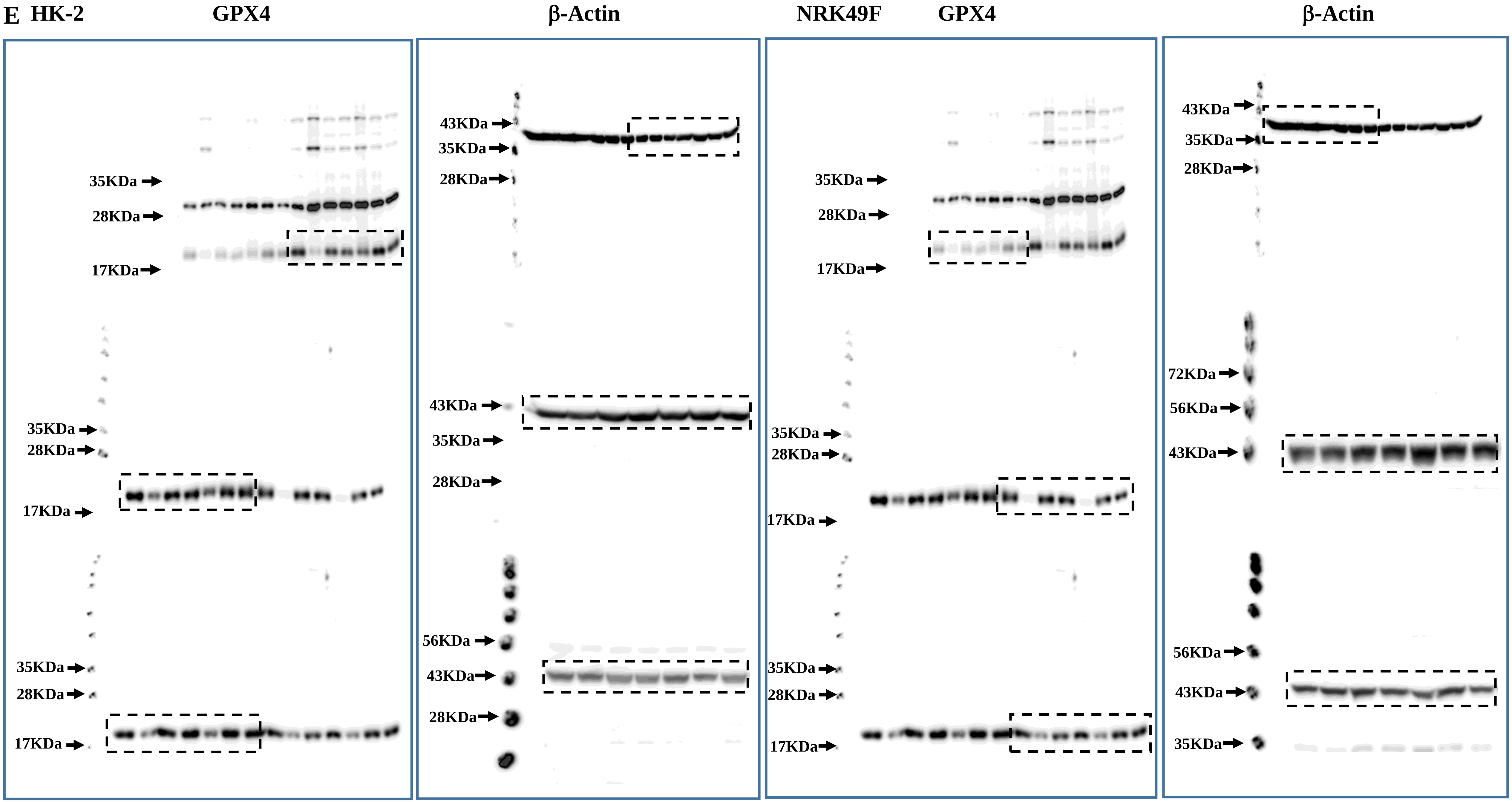

Fig 8 – raw data

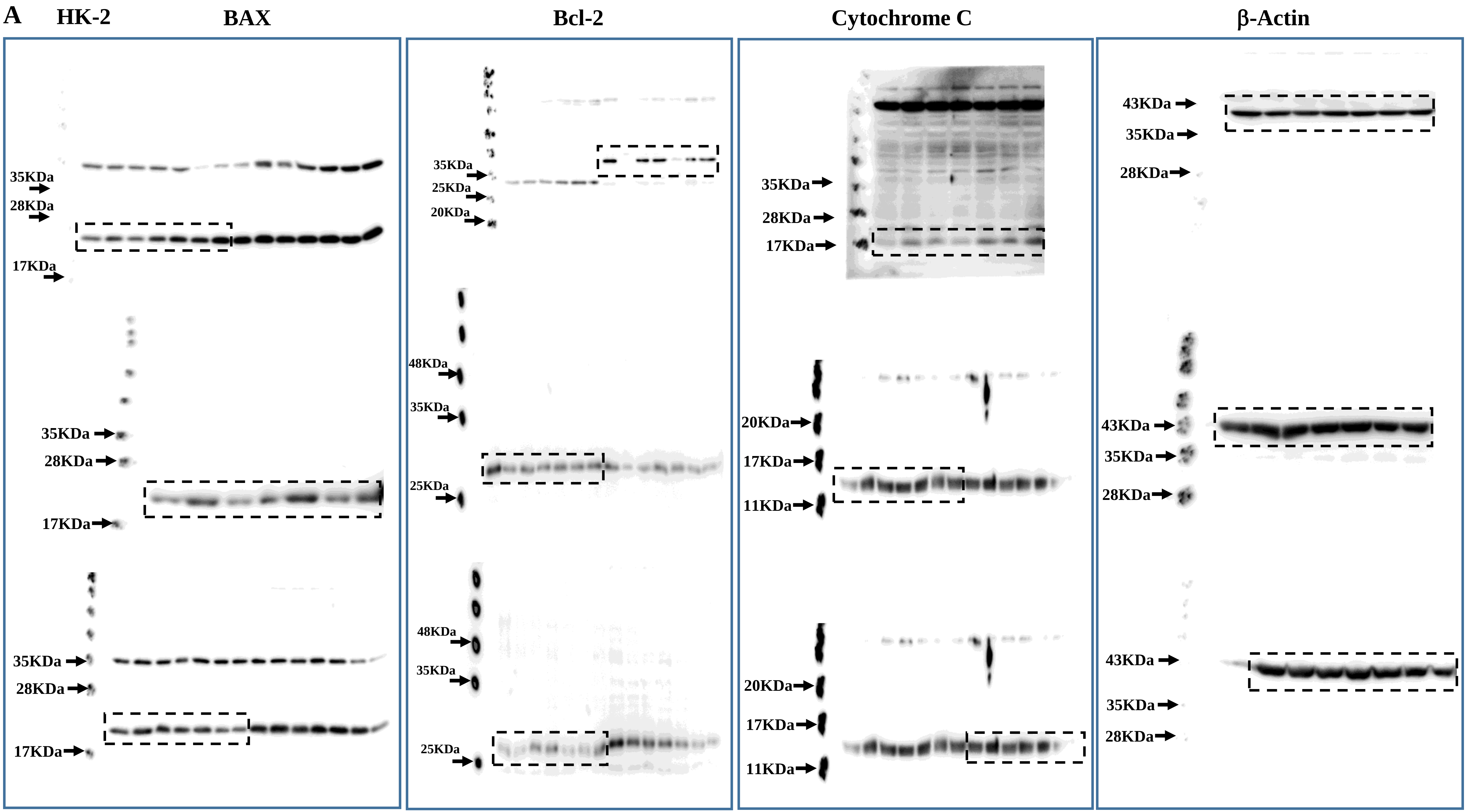

Fig 8 – raw data

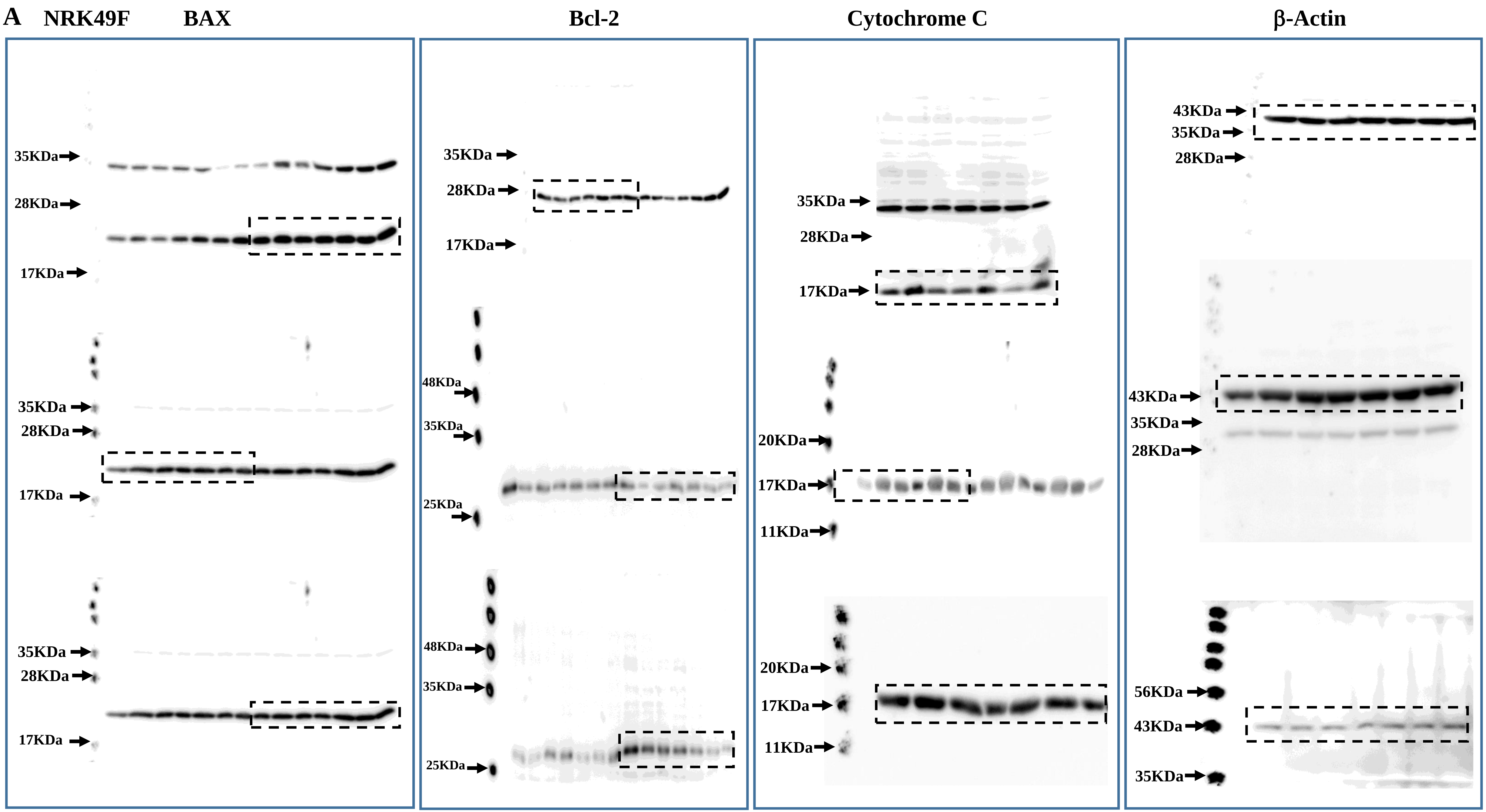

Fig 8 – raw data

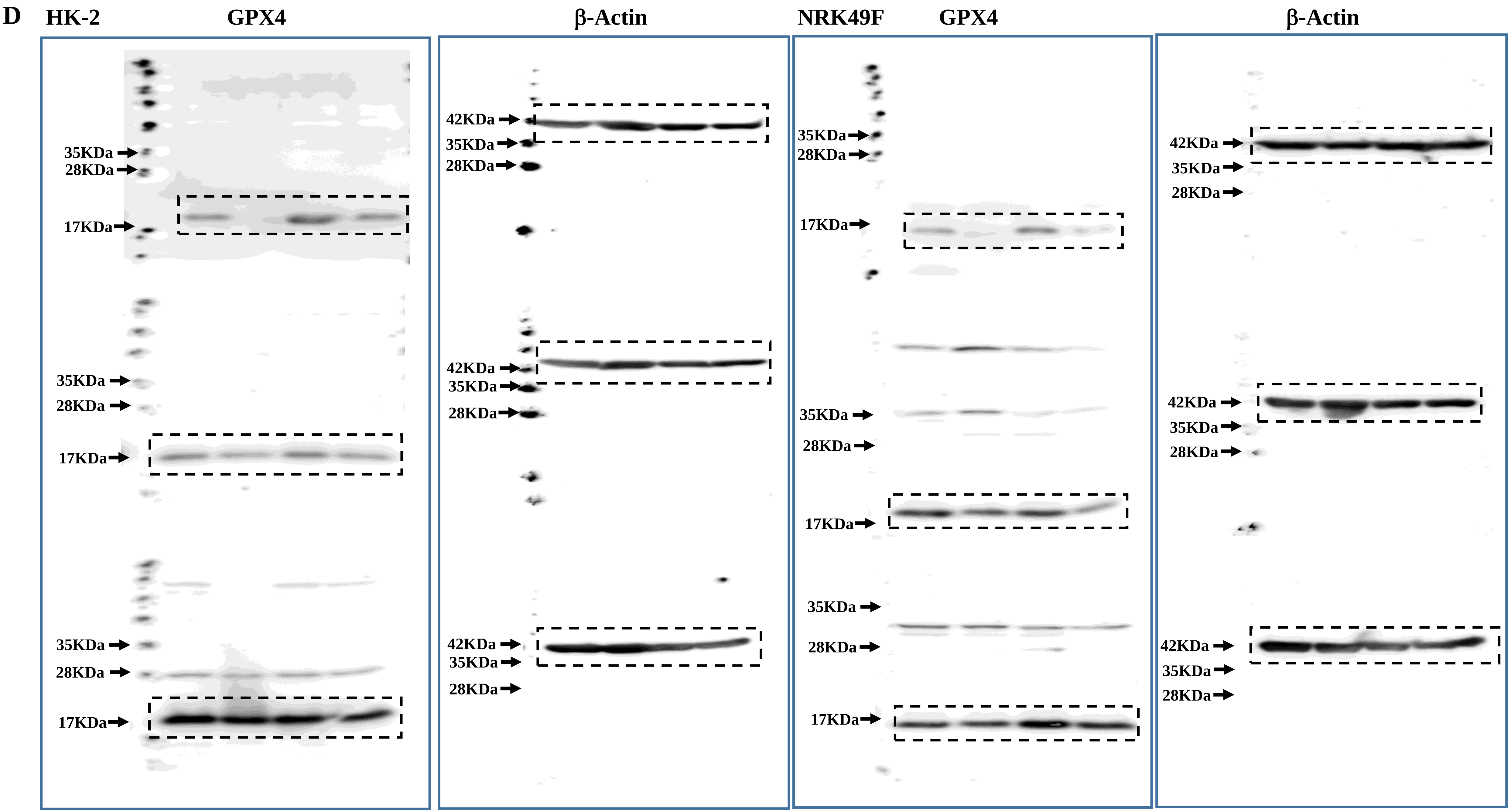

Suplemetary Fig 1 – raw data

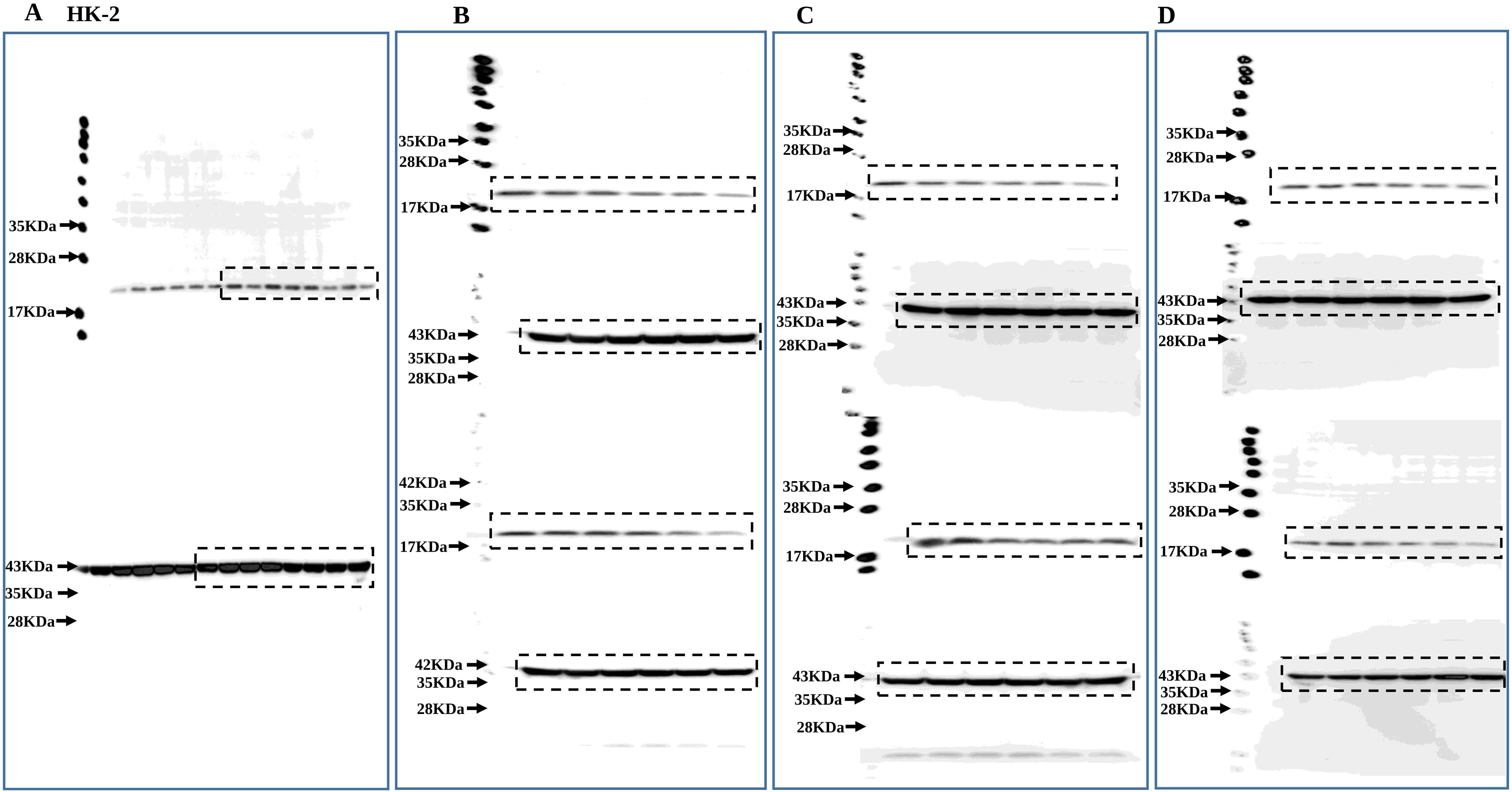

Supplement: Supplementary file 3 — Immunoblotting Raw Data [file 41419_2023_5601_MOESM3_ESM.pdf]
